# Activation of von Willebrand factor via mechanical unfolding of its discontinuous autoinhibitory module

**DOI:** 10.1038/s41467-021-22634-x

**Published:** 2021-04-21

**Authors:** Nicholas A. Arce, Wenpeng Cao, Alexander K. Brown, Emily R. Legan, Moriah S. Wilson, Emma-Ruoqi Xu, Michael C. Berndt, Jonas Emsley, X. Frank Zhang, Renhao Li

**Affiliations:** 1 Aflac Cancer and Blood Disorders Center, Children’s Healthcare of Atlanta, Department of Pediatrics, Emory University School of Medicine, Atlanta, GA USA; 2 Department of Bioengineering, Department of Mechanical Engineering & Mechanics, Lehigh University, Bethlehem, PA USA; 3 Biodiscovery Institute, School of Pharmacy, University of Nottingham, Nottingham, UK; 4 Faculty of Health Sciences, Curtin University, Perth, WA Australia

**Keywords:** Blood proteins, Single-molecule biophysics

## Abstract

Von Willebrand factor (VWF) activates in response to shear flow to initiate hemostasis, while aberrant activation could lead to thrombosis. Above a critical shear force, the A1 domain of VWF becomes activated and captures platelets via the GPIb-IX complex. Here we show that the shear-responsive element controlling VWF activation resides in the discontinuous autoinhibitory module (AIM) flanking A1. Application of tensile force in a single-molecule setting induces cooperative unfolding of the AIM to expose A1. The AIM-unfolding force is lowered by truncating either N- or C-terminal AIM region, type 2B VWD mutations, or binding of a ristocetin-mimicking monoclonal antibody, all of which could activate A1. Furthermore, the AIM is mechanically stabilized by the nanobody that comprises caplacizumab, the only FDA-approved anti-thrombotic drug to-date that targets VWF. Thus, the AIM is a mechano-regulator of VWF activity. Its conformational dynamics may define the extent of VWF autoinhibition and subsequent activation under force.

## Abstract (web summary)

Von Willebrand factor (VWF) is a large glycoprotein in the blood secreted from endothelial cells lining the blood vessel and activation of VWF leads to formation of VWF-platelet complexes or thrombi. Here authors use single-molecule force measurement, X-ray crystallography and functional measurements to monitor the activation of VWF via mechanical unfolding of the autoinhibitory module (AIM).

## Introduction

Virchow’s Triad describes the interplay between three broad categories of factors—blood, vessel, and flow—that contribute to thrombosis. Von Willebrand factor (VWF), a large, concatenated plasma glycoprotein^1^, is a canonical embodiment of such interplay. It is primarily secreted from endothelial cells lining the blood vessel, and it critically mediates hemostasis, thrombosis, and thromboinflammation by sensing and responding to changes in blood shear flow^2–4^. Under low shear conditions, plasma VWF is autoinhibited and does not bind glycoprotein (GP)Ibα, the major subunit of the platelet GPIb-IX complex. However, when VWF is either exposed to elevated shear or immobilized under flow, it experiences tension and subsequently exposes its A1 domain for binding to GPIbα and the platelet^5–7^. The binding transmits a signal into the platelet that leads to platelet aggregation and clearance^8–11^. Pathological binding of VWF to platelets in circulation could lead to microthrombosis, thrombotic thrombocytopenia, and sometimes organ failure^12,13^. Understanding the mechano-activation mechanism of VWF is key to elucidate the pathophysiology of thrombotic diseases and to develop safe anti-thrombotic therapeutics.

It has been documented for more than 30 years that under several conditions independent of flow change, VWF can overcome its autoinhibition and bind to GPIbα with high affinity. These conditions are present in some disease states, the most notable of which is type 2B von Willebrand disease (VWD). All reported type 2B mutations are located in the A1 domain or the flanking regions around A1^14^, suggesting that autoinhibitory elements are localized around A1. This is consistent with recent observations that global extension of VWF multimer under flow occurs before a local, tension-dependent activation of the A1 domain for GPIbα binding^5^. In addition, well-known activating agents, such as glycopeptide ristocetin and snake venom protein botrocetin, can also induce VWF binding to GPIbα in the absence of shear^15,16^. Ristocetin, but not botrocetin, mimics shear-dependent activation of VWF^17^. Although ristocetin is widely used in research and diagnostic tests, and the ristocetin-binding site in VWF has been mapped to include a proline-rich sequence following A1^18,19^, its mechanism of activation is not fully clear.

**Fig. 1:**
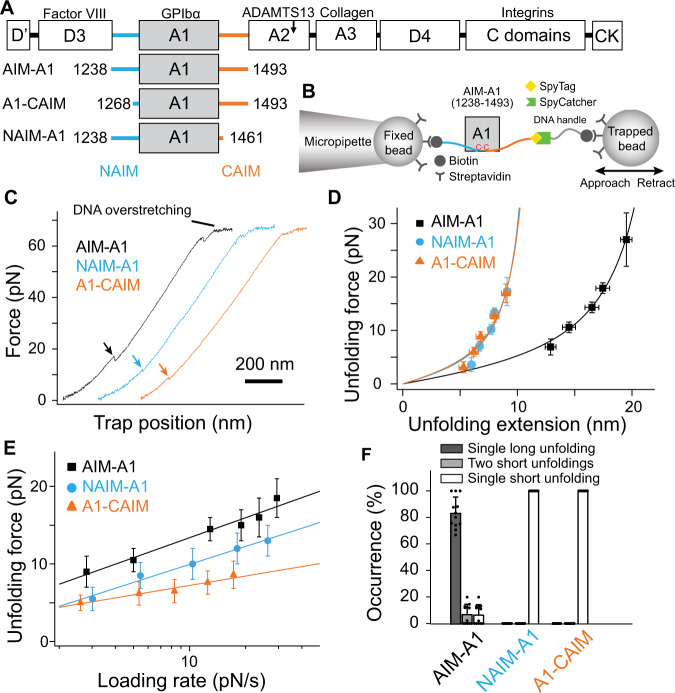
Cooperative unfolding of the discontinuous AIM. **A** Schematic of a VWF monomer, marked above with binding sites of related proteins, is aligned with various AIM-A1 fragments. NAIM and CAIM are colored cyan and orange, respectively. **B** Schematic of single-molecule optical tweezer apparatus in this study. The 1272–1458 disulfide bond is marked red in the A1 domain. **C** Representative force-extension traces of AIM-A1 (black), NAIM-A1 (cyan), and A1-CAIM (orange). The extension event in each trace is marked by an arrowhead. **D** Plots of unfolding force versus unfolding extension for noted AIM-A1 fragments and fits to the worm-like chain model. Force data are presented as mean values ± standard deviation, and extension data are presented as the peak of the Gaussian fit ± the full width at half maximum (FWHM) of Gaussian fit divided by the square root of counts. The data was obtained from *n* = 52, 80, and 75 biologically independent single-molecule tethers for AIM-A1, NAIM-A1 and A1-CAIM, respectively. **E** Plots of unfolding force versus loading rate for noted AIM-A1 fragments and fits to the Bell–Evans model. Unfolding force data are presented as the center of the tallest bin of the histogram ± one-half of the bin width. The data were obtained from *n* = 52, 80, and 75 biologically independent single-molecule tethers for AIM-A1, NAIM-A1, and A1-CAIM, respectively. **F** Average occurrence of a single long unfolding event (black bar), two short unfolding events (gray bar), and a single short unfolding event (white bar) during repeated cycles of extension and retraction. The constructs were pulled at 200 nm/s after relaxation under 1 pN for 1 s. Error bars represent standard deviation. *N* = 12, 17, and 19 biologically independent single-molecule tethers for AIM-A1, NAIM-A1, and A1-CAIM, respectively. Source data for (**D**–**F**) are provided in three worksheets of the Source Data file.

Crystal structures of individual domains of VWF show that the D’D3 assembly extends to residue 1237 and that the A2 domain starts at residue 1494^20,21^. The A1 domain is encompassed by the 1272–1458 disulfide bond and flanked by stretches of sequences (residues 1238–1271 and 1459–1493) that are O-glycosylated (Fig. 1). Truncating these flanking regions around the disulfide bond has yielded A1 fragments with disparate affinities for GPIbα. Their roles in modulating A1 binding have been speculated over the years, albeit without definitive evidence^19,22–27^. Except for a few residues close to the disulfide bond, these flanking sequences are not resolved in crystal structures of the A1 domain^28–30^. Our recent characterization of A1 fragments with differential affinities for GPIbα suggests that both N- and C-terminal flanking sequences cooperatively provide hydrogen–deuterium exchange protection on many residues in A1, particularly the β3α2 loop as a part of the GPIbα-binding site, and thus may constitute an autoinhibitory module (AIM)^19,27^.

Here, we report that the discontinuous AIM does resist tensile force, and cooperatively unfolds above a certain threshold of force to expose A1. We provide additional evidence that links disruption of the AIM with an increase of the A1 affinity for GPIbα under pathologically relevant conditions. Finally, the AIM could be targeted effectively by a recently developed antithrombotic agent.

## Results

### The AIM unfolds under tensile force, as a single unit

To detect and determine mechanical properties of the AIM under force, a recombinant AIM-A1 protein (containing VWF residues 1238–1493) with N-terminal biotin and a C-terminal SpyTag sequence^31^ was affixed to a SpyCatcher-biotin DNA handle, and trapped between two streptavidin-coated beads in an optical trap (Fig. 1A, B, Supplementary Fig. 1). The trapped construct was exposed to repeated cycles of extension and retraction. The AIM-A1 construct consistently showed an abrupt extension around 10–20 pN (Fig. 1C, Supplementary Fig. 2). This extension event could not be attributed to the unfolding of the A1 domain, which would have required rupture of its encompassing 1272–1458 disulfide bond and a rupture force greater than 100 pN^32,33^. Fitting extension traces of AIM-A1 to the worm-like chain (WLC) model^34^ yields a contour length of 26.6 ± 0.5 nm, suggesting that the underlying unfolding event involves approximately 67 residues, which is remarkably close to the length of both flanking sequences in the AIM-A1 construct (N-terminal 34, C-terminal 32) (Fig. 1D, Supplementary Fig. 3; Table 1). Replacing the DNA handle with a polyethylene glycol handle produced similar magnitudes of unfolding forces and extensions, indicating that, contrary to a recent report^35^, the DNA handle did not bind the A1 domain in this study and interfere with unfolding (Supplementary Fig. 4).

**Table 1:** Single-molecule force spectroscopy parameters associated with unfolding events of various constructs and additives.

Construct/Additive	Contour length *L*_*C*_ (nm)^a^	Persistence length *L*_*p*_ (nm)^a^	Unstressed unfolding rate ${k}_{u}^{0}$ (s^−1^)^b^	Barrier position ${\gamma }_{u}$ (nm)^b^
AIM-A1	26.6 ± 0.5	0.60 ± 0.05	0.074 ± 0.021	1.10 ± 0.10
A1-CAIM	13.1 ± 0.9	0.72 ± 0.17	0.093 ± 0.033	2.34 ± 0.29
NAIM-A1	12.9 ± 1.0	0.78 ± 0.22	0.154 ± 0.024	1.22 ± 0.09
AIM-A1 H1268D	17.7 ± 0.9	0.67 ± 0.11	0.288 ± 0.052	0.84 ± 0.12
AIM-A1 R1341Q	14.5 ± 0.3	1.32 ± 0.09	0.27 ± 0.12	1.15 ± 0.29
AIM-A1 with 6G1	14.6 ± 1.0	0.76 ± 0.19	0.10 ± 0.05	1.48 ± 0.28
A1-CAIM with 6G1	13.0 ± 0.6	1.08 ± 0.15	0.096 ± 0.008	3.38 ± 0.08
NAIM-A1 with 6G1	14.2 ± 1.4	0.95 ± 0.37	0.247 ± 0.069	1.17 ± 0.18
AIM-A1 with CR1	27.9 ± 2.2	0.37 ± 0.07	0.133 ± 0.030	0.94 ± 0.10
AIM-A1 with VHH81	25.3 ± 0.8	0.56 ± 0.07	0.0061 ± 0.0032	1.47 ± 0.14

^a^Contour length and persistence length are fitted worm-like chain model parameters. Uncertainties are the standard error of the fits.

^b^Unstressed unfolding rate and barrier position are fitted Bell–Evans model parameters. Uncertainties are the standard error of the fits.

While about 80–90% of force pulling cycles produced a single large extension event, a small percentage produced either one (5–15%) or two (~5%) smaller, separate extension events (Fig. 1F, Supplementary Fig. 2). Truncated “AIM-less” constructs, A1-CAIM (containing VWF residues 1268–1493) or NAIM-A1 (1238–1461), showed only one small extension event with contour lengths of 13.1 ± 0.9 and 12.9 ± 1.0 nm, respectively (Fig. 1D, F, Supplementary Fig. 3; Table 1). As small extension events in pulling A1-CAIM or NAIM-A1 closely resemble those small extension events in AIM-A1, each small extension should correspond to the unfolding of either NAIM or CAIM. More importantly, the large extension event in most AIM-A1 pulling traces should correspond to the concurrent unfolding of both NAIM and CAIM. In other words, the NAIM and CAIM cooperatively form a single structural unit that unfolds together. Not surprisingly, as shown in fits to the Bell–Evans model^36,37^, both NAIM-A1 and A1-CAIM exhibited lowered unfolding forces compared to AIM-A1 at all loading rates (Fig. 1E). Moreover, the NAIM unfolding force is higher than the CAIM unfolding force, in agreement with previous reports of the influence of NAIM residues on both GPIbα-A1 bond kinetics and platelet accumulation^25,38,39^. Nonetheless, both NAIM and CAIM contribute to the force resistance of AIM-A1.

It is noteworthy that refolding events were observed in only 9.6% of relaxation traces of AIM-A1. In cases where refolding was not apparent, AIM unfolding still occurred in subsequent pulls, indicating that refolding of AIM did happen when the molecule was relaxed at the present minimum hold force of 0.5 or 1 pN (Supplementary Fig. 5). The frequency of apparent refolding events decreased to 4.5% in NAIM-A1 and 3.1% in A1-CAIM, suggesting that refolding was more difficult or slower in these constructs. Overall, these results suggest that similar to refolding of the A2 domain^40^, AIM refolding is a relatively slow process and requires relaxation at low force for an extended time, most likely seconds. The AIM is likely metastable and has low folding free energy.

**Fig. 2:**
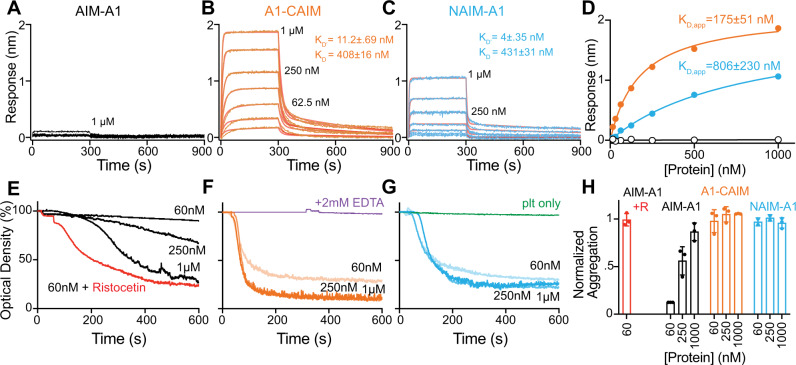
Truncation of either NAIM or CAIM activates A1. **A**–**C** Sensorgrams of VWF fragments binding to immobilized GPIbα-LBD. VWF fragments were serially diluted from 1 μM to 15.6 nM. Curves were fitted to a heterogenous-ligand binding model that accommodates two affinity states of A1. *K*_D’_ is the equilibrium dissociation constant of A1 binding to LBD with a higher affinity, while *K*_D_ is that with a lower affinity. Error is the standard deviation of the fitted rate constant. Fitting traces are in red, residuals are reported in Supplementary Fig. 6C. **D** Steady-state analysis of binding interactions between VWF fragments and GPIbα. Values from binding data shown in **A**–**C** are plotted and fit to a hyperbola. Apparent *K*_D_ is reported as mean ± 95% confidence interval. **E**–**G** Representative washed platelet aggregation traces of **E** AIM-A1, **F** A1-CAIM, and **G** NAIM-A1. 60 nM AIM-A1 + 1.5 mg/mL ristocetin is shown as a red trace in (**E**). The addition of 2 mM EDTA before the addition of VWF fragment A1-CAIM is shown as a purple trace in (**F**). Platelet only control is shown as a green trace in (**G**). **H** Extent of platelet aggregation at 600 s normalized to AIM-A1 at 60 nM with 1.5 mg/mL ristocetin. Data are means ± standard deviation, *n* = 3 per condition. Source data are provided in a worksheet of the Source Data file.

The importance of both NAIM and CAIM in shielding A1 from the ligand-binding domain (LBD) of GPIbα (residues 1–290) is made apparent by bulk binding measurements with AIM-less proteins. The AIM-A1 protein, at up to 1 µM concentration, showed little binding towards immobilized LBD, which is consistent with an apparent *K*_D_ of 32 μM previously reported for a glycosylated 1238–1471 fragment^41^. In comparison, either AIM-less protein showed a markedly higher apparent affinity for the LBD, indicating that removal of either half of the AIM exposes A1 (Fig. 2A–D, Supplementary Fig. 6). Binding sensorgrams were best fit to a two-phase association and dissociation, suggesting that A1 binding to LBD is likely more complex, with A1 adopting high- and low-affinity states^42,43^. Partial truncation of both NAIM and CAIM (tAIM-A1, containing VWF residues 1261–1472) also yielded an active A1 fragment with a similar apparent affinity as AIM-less proteins^19^ (Supplementary Fig. 6). Furthermore, at 60 nM, the normal physiological concentration of plasma VWF, both A1-CAIM and NAIM-A1, but not AIM-A1, induced significant aggregation of washed platelets, the kinetics of which exceeded aggregation induced by 60 nM AIM-A1 with 1.5 mg/ml ristocetin (Fig. 2E–H). The aggregation was inhibited by the addition of EDTA, suggesting that these A1 proteins triggered GPIb-IX signaling and activated integrin αΙΙbβ3 binding to fibrinogen^44^ (Fig. 2F). Interestingly, at higher concentrations, AIM-A1 could also induce aggregation, albeit at lower and slower responses than AIM-less proteins (Fig. 2E, H). Overall, these results demonstrate that the AIM constitutes a specific, cooperative shielding mechanism that is mechanically removable. Truncation of either part of the AIM, simulating mechanical separation of the AIM from A1, similarly exposes A1 for GPIbα binding and subsequent platelet aggregation.

### Type 2B VWD mutations destabilize or disrupt the AIM

**Fig. 3:**
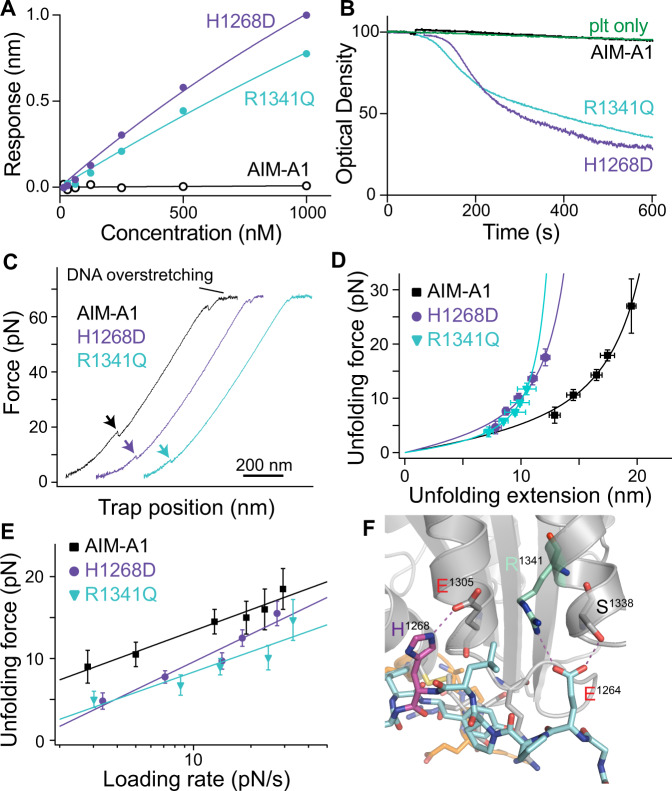
Type 2B VWD mutations alter AIM unfolding and A1 activity. **A** Steady-state analysis of WT AIM-A1, H1268D, and R1341Q to immobilized GPIbα. Protein was serially diluted from 1 μM to 15.6 nM and fit a hyperbola. **B** Washed platelet aggregation responses to 60 nM H1268D (purple), R1341Q (aquamarine), or WT (black). Resting washed platelets are shown in green. **C** Representative force-extension traces of H1268D (purple), R1341Q (aquamarine), and WT AIM-A1 (black). The extension event in each trace is marked by an arrowhead. **D** Superimposed plots of unfolding force versus unfolding extension data and their fits to the worm-like chain model. Force data are presented as mean values ± standard deviation, and extension data are presented as the peak of the Gaussian fit ± the FWHM of Gaussian fit divided by the square root of counts. The data were obtained from *n* = 52, 37, and 42 biologically independent single-molecule tethers for AIM-A1, H1268D, and R1341Q, respectively. **E** Regression of most probable unfolding forces fits the Bell–Evans model. Unfolding force data are presented as the center of the tallest bin of the histogram ± one-half of the bin width. The data were obtained from *n* = 52, 37, and 42 biologically independent single-molecule tethers for AIM-A1, H1268D, and R1341Q, respectively. Source data for **D**, **E** are provided in two worksheets of the Source Data file. **F** Structure of AIM-A1/VHH81 with highlighted interactions between H1268 (purple) to E1305 and R1341 (aquamarine) to E1264.

To test if type 2B VWD mutations alter mechanical properties of the AIM, two mutant AIM-A1 proteins bearing representative mutations (H1268D and R1341Q) were generated and characterized (Supplementary Fig. 7A). Patients with these mutations present with bleeding, thrombocytopenia, and a loss of high molecular-weight VWF^14,45^. Previously published crystal structures of A1 indicate a hydrogen bond between H1268 and E1305^30^, but no apparent interactions involving the side chain of R1341. As expected, both H1268D and R1341Q showed increased affinities towards the LBD than wild-type AIM-A1, although their affinities are weaker than those of AIM-less proteins (Fig. 3A, Supplementary Fig. 7B–E). Like AIM-less proteins, both mutants were able to spontaneously aggregate washed platelets at 60 nM (Fig. 3B). In single-molecule force measurements, H1268D showed a significant reduction in unfolding force at all loading rates compared to wild-type, with a large reduction in unfolding extension; R1341Q showed a single, short extension in the majority of traces (~90%) and one long extension event in the others (~10%), indicating the disruption of the cooperative AIM therein (Fig. 3C, D, Supplementary Fig. 2; Table 1). In most traces, the short extension had a low unfolding force, suggesting that it is due to the unfolding of the CAIM and that the NAIM is disrupted in this mutant (Fig. 3D, E, Supplementary Fig. 3). Overall, these results indicate that H1268D and R1341Q activate A1 by destabilizing or disrupting the AIM, and suggest that other type 2B VWD mutations could activate A1 in a similar manner.

### Disruption of the AIM-A1 interface by 6G1, a ristocetin-mimicking antibody

**Fig. 4:**
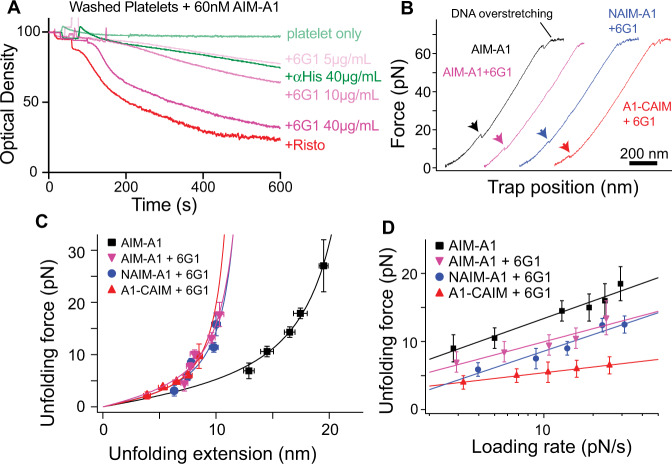
Disruption of AIM cooperativity by 6G1 activates A1. **A** Washed platelet aggregation in response to 1.5 mg/mL ristocetin (red), various concentrations of mAb 6G1 (pink), or anti-His-tag mAb (dark green) with 60 nM AIM-A1. Resting washed platelets are shown in light green. It should be noted the relative molarity for activation of 60 nM AIM-A1 is considerably lower for 6G1 compared to ristocetin, approximately 300 nM and 730 μM, respectively. **B** Representative force-extension traces of AIM-A1 alone (black) and AIM-A1 (pink), NAIM-A1 (blue), and A1-CAIM (red) in the presence of 30 µg/mL 6G1. The extension event in each trace is marked by an arrowhead. **C** Superimposed plots of unfolding force versus unfolding extension data and their fits to the worm-like chain model. Force data are presented as mean values ± standard deviation, and extension data are presented as the peak of the Gaussian fit ± the FWHM of Gaussian fit divided by the square root of counts. The data were obtained from *n* = 52, 58, 42, and 51 biologically independent single-molecule tethers for AIM-A1, AIM-A1 + 6G1, NAIM-A1 + 6G1, A1-CAIM + 6G1, respectively. **D** Regression of most probable unfolding forces fit the Bell–Evans model. Unfolding force data are presented as the center of the tallest bin of the histogram ± one-half of the bin width. The data were obtained from *n* = 52, 58, 42, and 51 biologically independent single-molecule tethers for AIM-A1, AIM-A1 + 6G1, NAIM-A1 + 6G1, A1-CAIM + 6G1, respectively. Source data for (**C**, **D**) are provided in two worksheets of the Source Data file.

Since ristocetin tends to flocculate proteins^46^ and may cause technical issues in single-molecule force measurements (Supplementary Fig. 4B), monoclonal antibody 6G1 was utilized in its place. 6G1 binds residues 1463–1472, a linear epitope in the CAIM, which overlaps with the ristocetin-binding sequence^19,47^ (Supplementary Fig. 8). In platelet-rich plasma (PRP), 6G1 could hinder ristocetin-induced platelet aggregation, owing to its competition with ristocetin binding^47^ (Supplementary Fig. 9A). Although 6G1 was unable to induce full platelet aggregation with plasma VWF, it dose-dependently induced aggregation of washed platelets incubated with 60 nM AIM-A1 (Fig. 4A, Supplementary Fig. 9B, C). The extent of platelet aggregation induced by 6G1 was significantly greater than that by anti-His-tag antibody at the same concentration, confirming that the effects of 6G1 on platelets were due to more than its divalent structure. Upon addition of 6G1 to the optical trap, most pulling traces of AIM-A1 showed a single smaller extension event with a lower unfolding force (Supplementary Fig. 2, Fig. 4B, C). The unfolding force of AIM-A1 with 6G1 is similar to that of NAIM-A1 with 6G1 but not A1-CAIM with 6G1, suggesting that 6G1 treatment disrupts folding of CAIM and/or its cooperativity with NAIM (Fig. 4D). In contrast to 6G1, a conformation-dependent monoclonal antibody CR1 that binds A1 and inhibits ristocetin-induced platelet aggregation^47^ did not alter the mechanical property of the AIM (Supplementary Figs. 3 and 10). Overall, these results indicate that the AIM can be disrupted by displacement of the CAIM through binding to 6G1. Since the 6G1 epitope overlaps significantly with the ristocetin-binding site^19^, ristocetin may activate A1 by disrupting the AIM in a similar manner.

### VHH81 binds to the NAIM and impedes AIM-A1-induced platelet aggregation

As both type 2B mutations and the antibody 6G1 activate A1 binding to GPIbα by destabilizing the AIM, we tested next if an exogenous factor can stabilize the AIM and inhibit A1 binding. Caplacizumab (ALX-0081) was recently approved by the FDA to treat thrombotic thrombocytopenic purpura (TTP), a disease characterized by the presence of active ultra-large VWF multimers due to insufficient ADAMTS13 activity^48^. Caplacizumab binds to VWF and blocks its interaction with platelet GPIbα^49^, but its mode of inhibition has not been elucidated. Caplacizumab is composed of two copies of the nanobody PMP12A2h1 (designated as VHH81 in this paper) linked by a tri-alanine sequence^49^. In this study, monomeric recombinant VHH81 was produced in bacteria and it bound purified VWF and plasma-derived VWF with ~20-nM affinity (Supplementary Fig. 11). Consistent with previous reports^49,50^, VHH81 dose-dependently inhibited ristocetin-induced binding of AIM-A1 to platelet GPIb-IX and platelet aggregation (Supplementary Fig. 12).

**Fig. 5:**
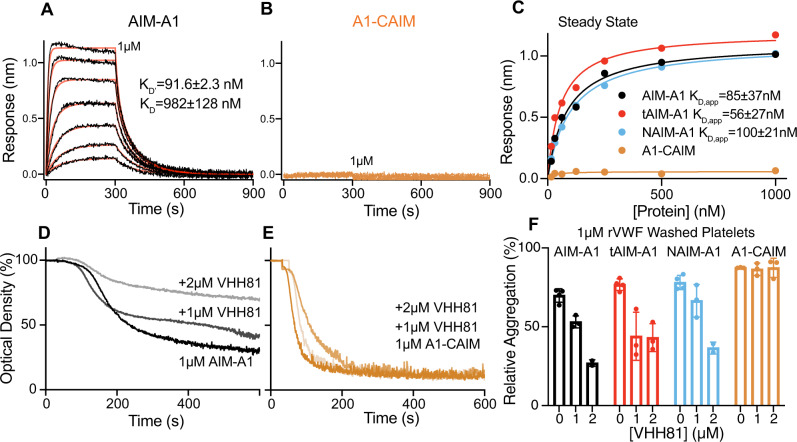
VHH81 binds to the NAIM to inhibit the A1-GPIbα interaction. Sensorgrams of immobilized AIM-A1 (**A**) or A1-CAIM (**B**) binding to serial dilutions of VHH81 from 1 μM to 15.6 nM. Global curve fits are displayed in red over binding traces. Fitted equilibrium dissociation constants are shown as globally fitted value ± standard deviation of the fit. No observable binding response was detected for A1-CAIM. **C** Steady-state binding curves of VWF constructs to VHH81. Binding data are shown in (**A**, **B**) and Supplementary Fig. 14 are plotted and fit to a hyperbola. tAIM-A1 comprises residues 1261–1472. Washed platelet aggregation responses to AIM-A1 (**D**) and A1-CAIM (**E**) pre-incubated with 0, 1, or 2 μM VHH81. **F** Comparison of relative inhibition of washed platelet aggregation using various concentrations of VHH81. In all cases, 1 μM VWF fragments were used. Data are means ± standard deviation, *n* = 3 per condition. Source data are provided in a worksheet of the Source Data file.

Through binding of various truncated AIM-A1 proteins with a FLAG-tagged VHH81, the binding epitope of VHH81 was mapped to include VWF residues 1261–1267 as VHH81 bound with high affinity to tAIM-A1 (containing VWF residues 1261–1472) and other NAIM-containing proteins, but not A1-CAIM (Fig. 5A–C, Supplementary Figs. 13 and 14). Consistently, VHH81 could impede aggregation of washed platelets induced by the aforementioned AIM-A1 proteins except for A1-CAIM (Fig. 5D–F, Supplementary Fig. 15).

**Fig. 6:**
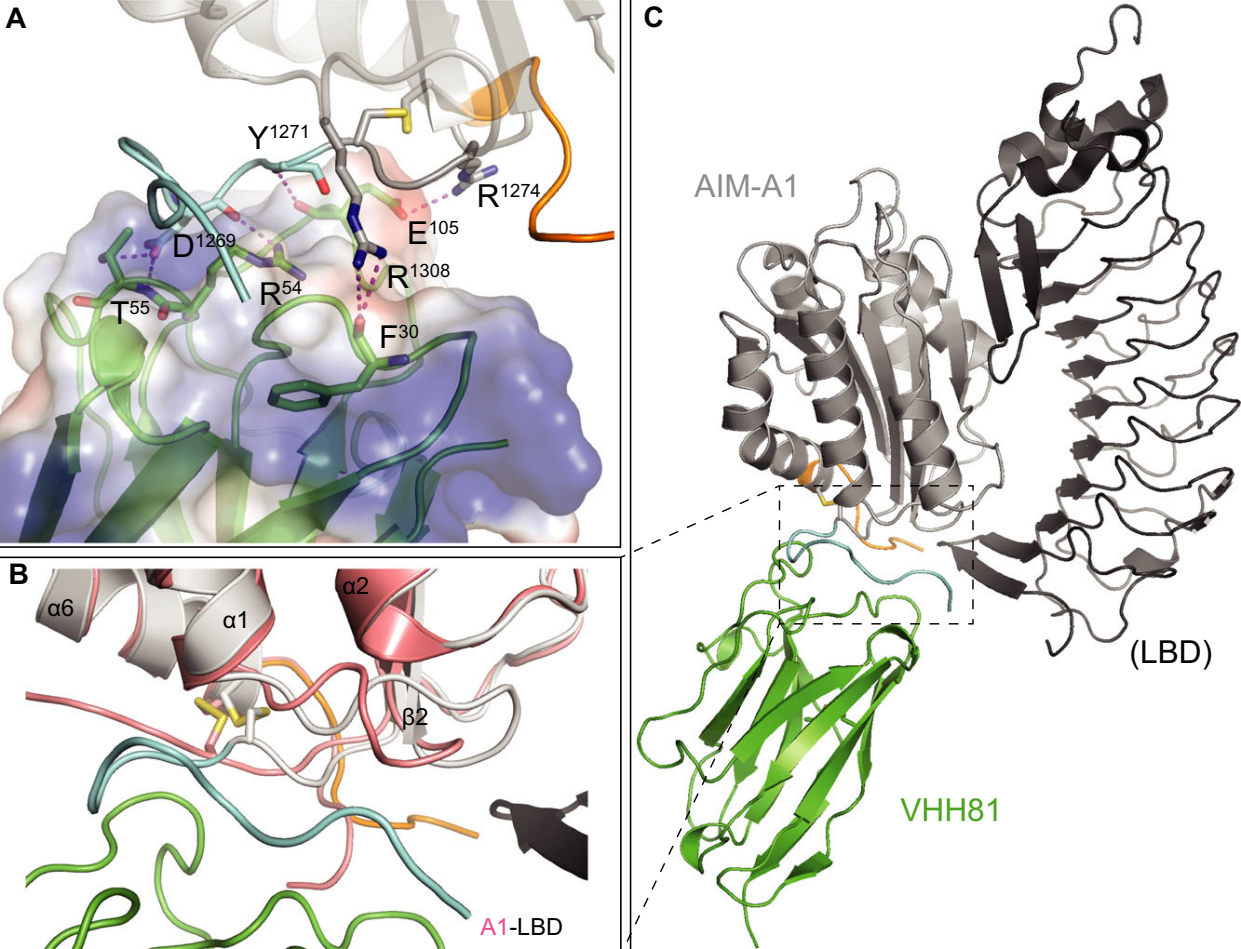
Crystal structure of the AIM-A1/VHH81 complex. **A** Co-crystal of VHH81 with AIM-A1 (Protein data bank (PDB) ID: 7A6O [10.2210/pdb7A6O/pdb]). The nanobody is shown in green overlaid with electrostatic surface potential. AIM-A1 is shown in gray with NAIM in cyan and CAIM in orange. Specific contacts of VHH81 to VWF are labeled. For instance, VWF residue R1308 forms a hydrogen bond with the backbone carbonyl of F30 in CDR1, both backbone and side-chain of D1269 make contacts with R54 and T55 in CDR2, residue R1274 forms a salt bridge to the side chain of E105, and the amide of Y1271 forms a hydrogen bond with the carbonyl of E105 in CDR3. **B** Conformational difference around the AIM between binding of VHH81 and GPIbα. Structures of the AIM-A1/VHH81 complex and the A1/LBD complex (PDB: 1SQ0 [10.2210/pdb1SQ0/pdb]) are superimposed by the shared A1 domain. Note that the α1β2 loop in A1 (colored salmon) in complex with LBD (black) is rotated upwards compared to the same loop in AIM-A1 (gray) in complex with VHH81 (green). In addition, resolved NAIM (cyan) and CAIM (orange) residues in AIM-A1 take on different conformations from those in A1. Some of them would be in steric hindrance with N-terminal residues in the LBD. **C** Overview of the two superimposed complexes. For clarity, the A1 domain in the A1/LBD complex is not shown. The dashed box outlines the interface area as highlighted in (**B**). Note that unresolved residues in the NAIM and CAIM would occupy the space surrounding the secondary GPIbα-binding site in the A1 domain.

To further characterize the interaction of VHH81 with AIM-A1, the crystal structure of a complex of VHH81 with a VWF fragment 1238–1481 was determined to 2.1-Å resolution (Fig. 6, Supplementary Fig. 16; Supplementary Table 1; Supplementary Video 1). In the structure, many residues, including residues 1262–1267 and some in the A1 domain, are in direct contact with all three complementarity-determining regions (CDR) loops of VHH81 (Fig. 6A). For instance, VWF residue R1274 forms a salt bridge to the side chain of E105 in CDR3, and residues 1262–1267 pack around Y32 in CDR1. Some CAIM residues also make contact with VHH81, such as the side chain of E1463 with backbone amides of R103 and A104 in CDR3. Also pertinent to this study is the hydrogen bond between side chains of E1264 and R1341, which has not been observed in any previous structures of A1 (Fig. 3F). Moreover, comparison of the AIM-A1/VHH81 complex structure to previously reported A1 structures, especially the A1/LBD complex structure^51^, reveals that the largest difference in VWF conformation lies in the α1β2 loop, as well as NAIM and CAIM residues, such that these residues appear to move away from the α1β2 loop upon binding of the LBD (Fig. 6B). In the AIM-A1/VHH81 complex, with residues, 1463–1466 in a position close to the α1β2 loop as shown in Fig. 6C, the unresolved residues beyond 1466 (i.e., residues 1467–1481) would clearly interfere with LBD binding to A1.

### VHH81 increases the mechanical stability of the AIM and is a shear-reversible antagonist of VWF

**Fig. 7:**
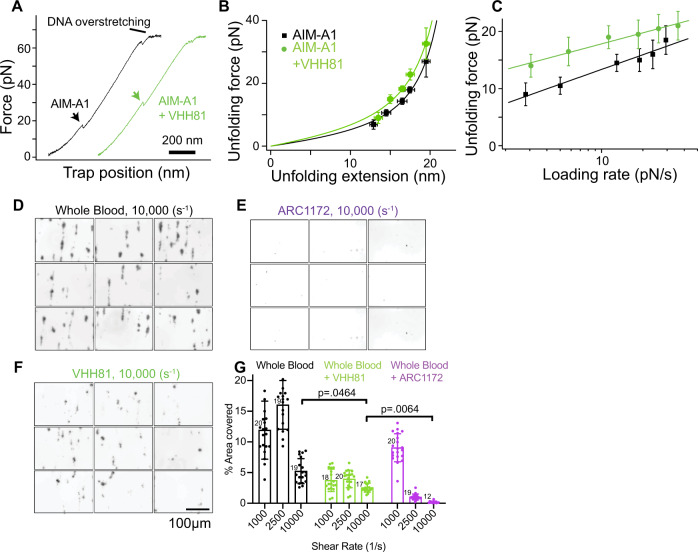
VHH81 acts as a shear reversible antagonist of A1-GPIbα by increasing the unfolding force of the AIM. **A** Representative force-extension traces of AIM-A1 unfolding with 1 µg/mL VHH81 (green) and without (black). The extension event in each trace is marked by an arrowhead. **B** Superimposed plots of unfolding force versus unfolding extension data and their fits to the worm-like chain model. Force data are presented as mean values ± standard deviation, and extension data are presented as the peak of the Gaussian fit ± the FWHM of Gaussian fit divided by the square root of counts. The data were obtained from *n* = 52 and 54 biologically independent single-molecule tethers for AIM-A1 and AIM-A1 + VHH81, respectively. **C** Regression of most probable unfolding forces fit the Bell–Evans model. Unfolding force data are presented as the center of the tallest bin of the histogram ± one-half of the bin width. The data were obtained from *n* = 52 and 54 biologically independent single-molecule tethers for AIM-A1 and AIM-A1 + VHH81, respectively. **D**–**F** Comparison of perfused whole blood platelet adhesion to collagen at 10,000/s shear rate. No inhibitors (**D**), ARC1172 (1 μM, **E**), or VHH81 (14 µg/mL, **F**) were mixed with recalcified blood before perfusion. The scale bar is 100 μm. **G** Comparison between platelet coverage was analyzed with a two-way ANOVA with mixed effects with Tukey’s multiple comparison correction. *P* values after multiple comparison corrections are displayed for relevant groups. Data are means ± standard deviation with *n* = number of fields analyzed per condition. Interaction *F*(4,155) = 42.12, shear rate *F*(2,155) = 67.17, treatment *F*(2,155) = 169.6. Source data for **B**, **C**, **G** are provided in three worksheets in the Source Data file.

To characterize the effect of VHH81 on the AIM under tension, single-molecule force measurement was performed to monitor AIM unfolding in the presence of 1 µg/mL VHH81. VHH81 binding increased significantly the unfolding force for AIM-A1 at all loading rates and did not alter its contour length (Fig. 7A–C, Supplementary Fig. 3; Table 1). The energy difference between the transition states could be calculated as Δ*G*_12_ = *k*_*B*_*T*⋅ln(*k*_1_/*k*_2_), where ${k}_{B}$ is the Boltzmann constant, *T* is the absolute temperature, and *k*_1_ and *k*_2_ are the unstressed unfolding rate constants of two A1 variants used for comparison, respectively. Using this equation and the unstressed unfolding rates from Table 1, the activation energy difference between AIM-A1 with and without VHH81 is estimated to be 2.5 *k*_*B*_*T*. Such enhanced unfolding activation energy may keep A1 masked under forces or shear stresses that would normally activate VWF. At the same time, such protection should be of a finite nature, since the VHH81-elevated force threshold could conceivably be still overcome by an even larger force, resulting in activation of VWF.

Platelet adhesion, activation, and aggregation over the collagen surface in a parallel-plate flow chamber have been extensively used to simulate blood clotting for hemostatic purposes^52^. At high shear rates, plasma VWF immobilized to the collagen surface becomes essential in mediating platelet adhesion^52^. Consistent with previous studies^49^, infusing VHH81 with whole blood over the collagen surface significantly inhibited platelet adhesion at various shear rates (Fig. 7D–G, Supplementary Figs. 17 and 18). Importantly, the inhibition by VHH81 was not complete, particularly at a shear rate of 10,000/s in which platelet adhesion is exclusively dependent on VWF^53,54^. In contrast, DNA aptamer ARC1172 or antibody 11A8, which directly blocks the VWF-GPIbα binding interface on A1 and LBD domains, respectively^19,55^, could completely abolish VWF-mediated platelet adhesion at high shear rates (Fig. 7G, Supplementary Fig. 17, 18). Since the 880-nM concentration of VHH81 used in this assay is much higher than its *K*_D_ for VWF and much higher than that used in vivo by typical dosing regimens^48,50^, VHH81 was likely bound to nearly all the A1 domain in plasma VWF (i.e., about 60 nM). This suggests that the incomplete inhibition by VHH81 is not due to incomplete binding to VWF. Thus, these results suggest that VHH81 inhibits the VWF-GPIbα interaction by a mechanism that protects the AIM from forces that would normally activate VWF.

## Discussion

**Fig. 8:**
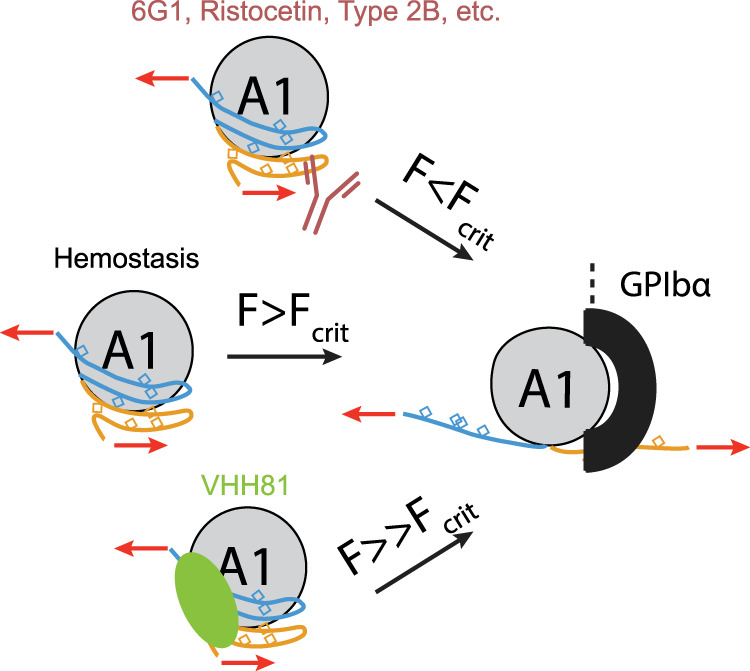
Molecular model of force-induced VWF A1 domain activation via dissolution of the AIM. During hemostasis, only above a critical force (*F*_crit_), will the AIM unfold to expose the A1 domain for GPIbα binding. VWF bearing type 2B mutations, binding to ristocetin, or mAb 6G1 lowers the critical unfolding force of the AIM, allowing GPIbα to bind under lower tensile forces. VWF bound to VHH81 is able to withstand forces that would normally activate A1 and increases the critical unfolding force of the AIM.

Coupling structural, functional and single-molecule analysis, we have provided the first evidence for a cooperative mechanical modulation of A1 binding by both halves of the discontinuous AIM. Deletion of either half of the AIM, the introduction of a type 2B VWD mutation at the AIM/A1 interface, or addition of a ristocetin-mimicking antibody that binds to CAIM residues results in the significantly decreased mechanical stability of the AIM and drastically increased activity of A1. These results suggest that widely documented factors of VWF activation, such as shear force, type 2B VWD mutations, and ristocetin, may share a common molecular mechanism—by destabilizing or disrupting the AIM and its shielding of the A1 domain (Fig. 8, Supplementary Video 2).

Numerous studies have reported that residues flanking the A1 domain could affect A1 binding to GPIbα and VWF activity^24,25,39^. A plethora of recombinant A1 fragments with variable lengths and glycosylation patterns displayed a wide range of affinities for GPIbα, spanning tens of micromolar to tens of nanomolar^22,24,29,56–58^. However, there has not been a coherent model to account for all the reported observations. For instance, it was postulated that an N-terminal flanking sequence, residues 1238–1260, interacts with and shields A1^38^. But this model could account for neither the sensitivity of residue 1472 polymorphism to ristocetin nor type 2B mutations in the C-terminal flanking region^18^. It was also postulated that residues immediately outside the 1272–1458 disulfide bond modulate A1 activity through their hydrogen bonding with A1^59^. While this model could potentially explain activation by force or some type 2B mutations, it could not explain why the recombinant 1261–1472 fragment binds platelets with high affinity and readily induces platelet aggregation^19^. Neither could it explain the type 2B-like activating effect by distal mutations such as T1255A^60,61^. Recently a model was proposed for a discontinuous AIM that consists of both N- and C-terminal flanking residues around A1^19^. This AIM model was supported by the reduced hydrogen–deuterium exchange rates in both halves of the AIM, as well as enhanced protection at the GPIbα-binding site in A1^19^. The cooperativity between NAIM and CAIM was postulated but lacked direct evidence. Relatedly, doubt was raised about the AIM as a distinct structural entity, as it was suggested that the flanking regions simply sterically occlude GPIbα binding^41^. In this study, we demonstrated that the AIM constitutes a single structural unit as it unfolds under tensile force mostly in a single extension event instead of separate unfolding events of NAIM and CAIM (Fig. 1F). Importantly, the contour length increase of 26.6 nm (Fig. 1, Table 1) corresponds to about 67 residues present in unstructured regions after the unfolding event, which most likely include both NAIM and CAIM. The unfolding force of the AIM is greater than the individual unfolding forces of NAIM and CAIM, providing additional evidence supporting the cooperativity of NAIM and CAIM. Moreover, type 2B mutations or addition of the ristocetin-like antibody also disrupted cooperative unfolding of the AIM, resulting in significantly lowered unfolding force and shortened contour length increase.

Responses of full-length VWF and its fragments to mechanical force have been studied using optical or magnetic tweezers as well as atomic force microscopy. Several mechano-responsive elements therein have been identified, including the D4 assembly and the A2 domain^40,62–65^. It is noteworthy that the unfolding force of the AIM is 15–20 pN, which is similar to that of the A2 domain. A recent study estimated the local tensile force necessary to activate A1 in full-length multimeric VWF as 21 pN^5^, which is in line with the unfolding forces of the AIM. The D’D3 assembly and A2 domain have been suggested to modulate A1 binding to GPIbα^66–69^. Considering the proximity of these domains to AIM and A1, it is not unreasonable to speculate they may also affect the mechanical stability of the AIM through their interactions. The details of such modulation await future investigation.

Our results have provided several insights on the structural basis for the cooperativity of the AIM. In the AIM-A1/VHH81 complex structure (Fig. 6), in which the AIM is stabilized, residues 1238–1261 and 1265–1481 are not resolved. They do not appear to assume a stable structure. Considering the large contour length of the AIM as well as the reduced hydrogen–deuterium exchange rates in some residues therein^19,27^, a likely scenario is that these residues are partially folded, but they interact specifically with each other to form a cohesive module. Certain mutations, such as at residues D1261 and T1255^26,60,61^, may conceivably disrupt these interactions, destabilize the AIM, and produce type 2B-like effects. On the other hand, although no direct contacts are observed between NAIM and CAIM residues that are resolved in the complex structure, many of them form hydrogen bonds and salt bridges with residues in the A1 domain, sometimes through a water molecule. Thus, in addition to NAIM and CAIM residues, those in the A1 domain may also contribute to the cooperativity in the AIM. It is not a coincidence that all of type 2B VWD mutations reported to-date are located at or near the AIM-A1 interface and should disrupt the elaborate interaction network thereof. In particular, our AIM-A1/VHH81 complex structure shows an interaction between residues 1264 and 1341 that has not been observed before (Fig. 3F). It is unknown if this interaction is present in AIM-A1 without VHH81 but could explain the activating effect of type 2B mutations at R1341. It may also provide structural evidence for the increase in force resistance of the AIM when bound to VHH81. Similarly, the AIM-A1 interface as observed in the complex structure illustrates the effects of many type 2B mutations such as P1337L and those of residues 1305, 1306, 1308, and 1309 at the base of the α1 helix or α1β2 loop could certainly disrupt the interactions between NAIM and A1. The exact residues responsible for cooperativity between NAIM and CAIM are subjects of future study and may yield new synthetic VWD mutations.

The interaction of platelet GPIbα with VWF through their respective LBD and A1 domains is critical to thrombus formation in many thrombotic diseases. It has been a target in the development of antithrombotic therapeutics for the last few decades^70^. Many competitive inhibitors that directly block the binding site in either LBD or A1, including monoclonal antibodies, conformationally constrained peptides, DNA aptamers, and snake venom derivatives, have been reported^71–76^. Since the GPIbα-VWF interaction is essential to primary hemostasis, as genetic deletion of either protein would result in a severe bleeding disorder^3,77^, pharmacological inhibition of the interaction may lead to side effects of severe bleeding. Caplacizumab is the first and to-date the only inhibitor of the GPIbα-VWF interaction that has been approved by the FDA^48,78^. TTP patients treated with caplacizumab showed a small risk of a bleeding event, mostly limited to epistaxis or gingival bleeding. The severity of these events was low and almost entirely resolved without intervention^48,78^. In this study, we show that VHH81 differs from all the previously reported inhibitors of the GPIbα-VWF interaction. It does not directly interfere with the GPIbα-binding site in A1 but rather binds to primarily NAIM residues. VHH81 stabilizes the AIM-A1 interface, as exemplified by the interaction between residues 1341 and 1264, and increases the unfolding force threshold for the AIM. In other words, the binding of VHH81 raises the shear threshold of VWF mechanoactivation (Fig. 8). These results could explain why at very high shear rates VHH81 could not completely abolish VWF-mediated platelet adhesion, whereas traditional antagonists such as ARC1172 could and thus would render VWF completely incapable of platelet capture at high shear rates as required for normal hemostasis (Fig. 7F). This critical difference may help explain the lack of major bleeding risk with caplacizumab. While inhibiting the GPIbα-VWF interaction always presents a risk of bleeding, our results suggest that the approach of targeting the AIM may be more productive with less impact on hemostasis than that of direct antagonism of the A1 domain.

As VWF is critical to primary hemostasis and also a number of thrombotic diseases, its level, size, and binding activity are tightly regulated. In this study, we have provided evidence supporting a model of the discontinuous AIM as the mechanism of VWF mechanoactivation. This model may also be applicable to other scenarios of VWF activation, including type 2B VWD and ristocetin-induced platelet aggregation. While the AIM can be destabilized or disrupted for an increase in VWF activity, it could also be stabilized with anti-thrombotic consequences. These observations suggest that modulation of the AIM, mechanically or thermodynamically, maybe a common mechanism for the regulation of VWF function. Furthermore, to the best of our knowledge, VHH81 is the first reported case by which a drug modulates the function of its target via mechanically linked allostery. As an increasing number of mechanosensors and mechanoreceptors are being identified and linked to a variety of diseases, the example of VHH81 suggests they could be likewise targeted for mechanical modulation and therapeutic purposes.

## Methods

### Materials

Ristocetin was purchased from MP Biomedicals. Most recombinant VWF fragments and type 2B mutants were expressed from baby hamster kidney cells as described^19,27^. Plasma-derived VWF was reconstituted in phosphate-buffered saline (PBS) from outdated lyophilized Humate-P (CSL-Behring). Monoclonal antibodies 6G1, CR1, and 11A8 have been described^19,47^. ARC1172^55^ was synthesized by Integrated DNA Technologies. Mammalian cells were maintained in DMEM/F12 culture media (ThermoFisher), with 10% Foundation-B fetal bovine serum (Gemini Bio-Products, Sacramento, CA), and 1% penicillin/streptomycin (ThermoFisher). Bovine serum albumin, fraction V was purchased from Fisher Scientific. Tobacco etch virus (TEV) protease was produced with plasmid pD2087 in BL21pRIL cells and purified as described^79^. The Human GPIb-IX complex was purified from outdated and deidentified leuko-reduced apheresis-derived platelets as described^80^.

### Construction of Expi293F-BirA cells

The gene encoding *E. coli* biotin ligase BirA was subcloned from vector pBIG5b^81^ using the EcoRI and XbaI sites and ligated into pcDNA3.1-Zeo(+) vector (Invitrogen). The resulting plasmid was transfected into Expi293F cells using Lipofectamine 3000 (ThermoFisher). Single clones were selected using 400 µg/mL zeocin (ThermoFisher). For biotinylation, Expi293F-BirA cells were transfected to express proteins bearing a BioTag (LNDIFEAQKIEWH) sequence in 10 μM biotin.

### Recombinant VWF and BioSpy-VWF fragments

For recombinant VWF fragment 1268-1493 (A1-CAIM), the encoding DNA fragment was amplified from the expression vector encoding 1238-1493-10His using primers IgK-1268F and 1268XbaStopR (all the primers are listed in Supplementary Table 1), and subcloned into the pcDNA3.1-Hygro(+) vector (Invitrogen) as an XbaI-NheI fragment. The resulting vector was subsequently transfected into Expi293F cells for stable protein expression and purification as described using a GE Healthcare Ni Sepharose excel column followed by size exclusion chromatography on a GE Healthcare HiLoad 16/600 Superdex 200 pg column^19,27^.

To clone BioSpy-VWF constructs, a decahistidine (10His) and SpyTag (AHIVMVDAYKPTK)^31^ sequence was appended to the C-terminus of VWF fragments using primers 1493F and 1493RSpyStop. Each gene fragment was ligated into the pBIG4a vector using SpeI and XhoI sites such that a consensus Kozak sequence, α1-antitrypsin signal sequence, and a BioTag was appended to the N-terminus^82^. The expression cassette was subsequently subcloned into pcDNA3.1-Hygro as a NheI-XhoI fragment. Type 2B constructs were generated by site-directed mutagenesis using primers EL003/EL004 for R1341Q and EL007/EL008 for H1268D. All DNA sequences were verified by sequencing.

Each pcDNA-BioSpy-VWF vector was transfected into adherent Expi293F-BirA cells using Lipofectamine 3000. Single clones were selected using 250 µg/mL hygromycin B (ThermoFisher). Stably expressing clones were adapted to SFM4-CHO UT (GE Healthcare) or FreeStyle F17 Expression media (ThermoFisher), supplemented with 8 mM l-Glutamine or 2× GlutaMAX (ThermoFisher) in 125-mL flasks (Thomson Instruments) or 50-mL spinning culture vessels (Corning). Cells were passed into a 250-mL flask or 500-mL spinning flask at 200,000–400,000 cells/mL and cultured for 7–10 days. Protein was purified from the media as previously described^27^. When needed, cell-free biotinylation was performed using the BirA500 kit from Avidity LLC (Aurora, CO). Excess biotin was removed by size-exclusion chromatography on a HiLoad 16/600 Superdex 200 pg column (GE). Subsequent fractions were tested for biotinylation by Western blot using Streptavidin IR-Dye680 (Licor) (1:2000), verified using anti-His-tag antibody 4E3D10H2/E3 (ThermoFisher) (1:2000) followed by secondary antibody IRDye 800CW Goat anti-Mouse IgG (Licor) (1:5000) and analyzed for purity by Coomassie stain. Purified protein was stored at −80 °C before use.

### Recombinant LBD of GPIbα

The gene fragment encoding human GPIbα residues His1-Arg290 was amplified from a GPIbα vector^83^ using primers GPIba_Biotag and GPIba290_2xFLAGstop to append a BioTag and a 2×FLAG tag to N- and C-termini, respectively. This fragment was ligated into a modified pcDNA3.1-Hygro vector, which contains a signal sequence followed by a 10His tag and the TEV protease cleavage sequence at the N-terminus, as a BamHI-XhoI fragment. Stably expressing clones were generated in *Expi*293F-BirA cells. The protein was purified using the same method as for VWF fragments.

### Construction and production of VHH81 nanobody

The sequence of VHH81 was obtained from international patent WO2011/067160 (clone PMP12A2h1) and its encoding DNA fragment was synthesized by Integrated DNA Technologies. For crystallization experiments, primers pD14_VHH81F and pD14_VHH81R were used to amplify a gene fragment encoding VHH81 with C-terminal hexahistidine (VHH81-6His), cloned into a modified pDEST14 vector^31^, and produced in the cytoplasm of SHuffle T7 Express cells. To induce expression in both cases, 0.4 mM IPTG was added to bacteria culture at OD_600_ of 0.9 at 30 °C. After 4-5 h, cells were centrifuged at 8,000 g for 20 min at room temperature and lysed with BugBuster with benzonase (Novagen/Sigma-Aldrich) according to the manufacturer’s directions. The lysate was centrifuged at 17,000 g and supernatant filtered by a Steriflip unit (Millipore). VHH81-6His was purified by Ni-affinity chromatography and gel filtration chromatography in PBS. Purified protein was flash-frozen in liquid nitrogen and stored at −80 °C until use.

To express Flag-VHH81 for BLI experiments, the gene fragment encoding VHH81 with an N-terminal FLAG tag, a C-terminal TEV protease cleavage sequence, and a 6His tag was cloned into the pET22b + plasmid and expressed in SHuffle T7 Express cells (New England Biolabs). To purify Flag-VHH81 from the periplasm, cell pellets were resuspended in 30 mM Tris-HCl, 20% sucrose, pH 8.0, at 80 ml per gram wet weight. EDTA was added dropwise to 1 mM. Cells were incubated on ice for 10 min with gentle agitation. The cell suspension was centrifuged at 8000 *g* for 20 min at 4 °C, and the pellet resuspended in the same volume of ice-cold 5 mM MgSO_4_. The cell suspension was incubated on ice for 10 min with gentle agitation. The suspension was centrifuged as before, and the protein in the supernatant was purified by Ni-affinity chromatography. Cleavage of the 6His tag by recombinant TEV protease (1 mg nanobody/125 μg protease) was performed overnight at 4 °C in PBS with 10% glycerol. The mixture was applied to a His-Trap column and the flow-through containing Flag-VHH81 collected and analyzed via western blot and ELISA. The lack of the 6His tag in purified Flag-VHH81 was verified via immunoblot with anti-His antibody 4E3D10H2/E3 at 1:2000 dilution or ELISA (Supplementary Fig. 13).

### Blood preparation

Human fresh whole blood was obtained from healthy donors via venipuncture into Vacutainer 3.2% sodium citrate tubes (BD). Written informed consent was obtained from participants before their inclusion in studies, and all procedures using donor-derived human blood and platelets were approved by the Institutional Review Board at Emory University.

### Platelet aggregometry

PRP and washed platelets were prepared from citrated whole blood as described^84^. For platelet aggregometry, washed platelets were resuspended in modified Tyrode’s buffer (134 mM NaCl, 2.9 mM KCl, 0.34 mM Na_2_HPO_4,_ 12 mM NaHCO_3_, 20 mM HEPES, 1 mM MgCl_2_) with 5 mM d-glucose. Platelets were recalcified with 5 mM CaCl_2_ and were normalized to 150,000/μL at 240 uL per cuvette. Recombinant AIM-A1 fragments were centrifuged at 100,000 g for 30 min at 4 °C, and the protein concentration was measured on a Nano-Drop (ThermoFisher) using the protein’s extinction coefficient^27^. After a stable baseline was established, AIM-A1 fragments were added to the platelet suspension. In tests of VHH81 inhibition, VHH81 was mixed with AIM-A1 fragments and then added to the platelet suspension. Alternatively, VHH81 was added to the PRP in the cuvette, followed 30 s later by the addition of 1.5 mg/mL ristocetin. In all cases, platelet samples before experiments were monitored for premature aggregation and activity verified at various time points by observing full aggregation after adding 60 nM AIM-A1 and 1.5 mg/mL ristocetin. Platelet aggregation was recorded in AGGRO/LINK software (Chrono-log, Havertown, PA), exported and normalized to initial optical densities of 100 manually. The extent of aggregation was measured as the optical density at 600 s.

### Bio-layer interferometry (BLI)

BLI experiments were performed on an Octet QK^e^ instrument (ForteBio) using black non-binding platelets (Greiner Bio-One, Monroe, NC) and manufacturer-supplied Data Acquisition software v11.1.1.19. Plate temperature was set to 23 °C and plate shaking to 1,000 rpm. Streptavidin sensors (for biotinylated LBD) or Ni-NTA sensors (for AIM-A1 fragments) were equilibrated in the kinetics buffer (KB, ForteBio) for at least 10 min prior to initiation of the experiment. All proteins were diluted in sample diluent (ForteBio) to minimize non-specific interactions. Equilibrated sensors were loaded with 15 µg/mL biotinylated LBD for 300 s, followed by a 120-s baseline. Sensors were then dipped into AIM-A1-containing wells for 300 s of association, followed by dissociation in KB for 300 s. To regenerate the sensors, the sensors were regenerated by 4 cycles of 5-s wash in 2 M NaCl, followed by 5 s in KB. Consistent LBD regeneration was evidenced by a return to baseline accumulation after the initial loading step at 0 nm. A reference sensor was included in all measurements whereby LBD was loaded to the sensor but VWF fragments were absent in wells. For binding to Flag-VHH81, VWF fragments were loaded to Ni-NTA sensors at a set threshold of 3 nm. Sensors were regenerated in 5-s cycles of 10 mM glycine, pH 1.6, and neutralized in KB. Sensors were reloaded with 10 mM NiCl_2_ for 60 s. Baseline subtraction of the loaded reference sensors was applied to all runs. The heterogeneous ligand, global curve fitting was performed on binding experiments using the Data Analysis HT software v11.1.1.39 (ForteBio). In this scheme, both high- and low-affinity A1 could bind to immobilized LBD,

$${A}^{{\prime} }+B\, \rightleftarrows\, A^{\prime} B$$

$$A+B\, \rightleftarrows\, {AB}$$

where *A*’ is the higher affinity A1. In this model, interconversion of *AB* to *A’B* was not accounted for as the binding was performed in the absence of force. Similarly, VHH81 binding is dependent on the structure of a discontinuous binding epitope spanning the NAIM and A1, where A1 could sample both states. Data was exported to Prism and the steady-state plots were fit to hyperbolic binding curve. Each set of experiments were repeated at least twice.

### Single-molecule force spectroscopy

Single-molecule force measurement was performed largely as described^85^. Briefly, the biotinylated VWF fragment (e.g., BioTag-1238–1493-10His-SpyTag) was immobilized on a streptavidin bead held by a fixed micropipette. SpyCatcher protein with a C-terminal Cys residue^31^, a kind gift from Dr. Mark Howarth, was coupled to a biotin-DNA handle of 802 bp, and then coupled to streptavidin beads of 2.0-μm diameter (Spherotech, Lake Forest, IL). For pulling experiments, the SpyCatcher-DNA handle bead, trapped and controlled by the optical tweezer, was brought to interact with the VWF fragment bead. The force measurement was performed at force-ramp mode with varying pulling speeds (50, 100, 200, 400, and 500 nm/s) in Tris-buffered saline (20 mM Tris, 150 mM NaCl, pH 7.5). The force-extension data were fitted to the WLC model

1 $$\frac{F(x)\cdot {L}_{p}}{{k}_{B}T}=\frac{1}{4}{\left(1-\frac{x}{{L}_{c}}\right)}^{-2}-\frac{1}{4}+\frac{x}{{L}_{c}},$$

where $F(x)$ is the applied force on the polymer, $x$ is the end-to-end distance, ${L}_{c}$ is the contour length, and ${L}_{p}$ is the persistence length of the polymer. Unfolding extension is defined as the increase in end-to-end distance between the point of unfolding and the point at which the force at unfolding is re-established. In order to find the most probable extension at various forces, the force-extension data were first binned by force. Next, the extension data within each bin was plotted as histograms to identify the peak extension (Supplementary Fig. 3A). Unfolding was also analyzed according to the Bell–Evans model, a theory first developed to describe the influence of an external force on the rate of molecular complex dissociation^36,37^ and has been applied later to study protein unfolding^40,85^. In this model, a pulling force, *f*, distorts the intramolecular potential of a protein complex, leading to lower activation energy and an increase in the unfolding rate *k*_*u*_*(f)* as follows

2 $${k}_{u}(f)=1/{t}_{u}(f)={k}_{u}^{0}\,\exp (\frac{f{\gamma }_{u}}{{k}_{B}T})$$

where ${k}_{u}^{0}$ is the unfolding rate constant in the absence of a pulling force, ${\gamma }_{u}$ the barrier position, *T* the absolute temperature, and ${k}_{B}$ the Boltzmann constant. For a constant loading rate *R*_*f*_, the probability for the unfolding of the complex as a function of the pulling force *f* is given by

3 $$p(f)={k}_{u}^{0}\,\exp (\frac{f{\gamma }_{u}}{{k}_{B}T})\exp \{\frac{{k}_{u}^{0}{k}_{B}T}{{\gamma }_{u}{R}_{{\rm{f}}}}[1-\exp (\frac{f{\gamma }_{u}}{{k}_{B}T})]\}$$

with the most probable unfolding force f*

4 $$f\ast =\frac{{k}_{B}T}{{\gamma }_{u}}\,{\mathrm{ln}}(\frac{{\gamma }_{u}}{{k}_{u}^{0}{k}_{B}T})+\frac{{k}_{B}T}{{\gamma }_{u}}\,{\mathrm{ln}}({R}_{{f}})$$

Bell–Evans model parameters ${k}_{u}^{0}$ and ${\gamma }_{u}$ were determined by fitting equation [4] to the plot of f* vs. ln(*R*_*f*_).

### Enzyme-linked immunosorbent assay (ELISA)

VWF and recombinant AIM-A1 fragments (6 μg/mL in PBS) were coated to high-binding half-area 96-well plates (Corning). VHH81 binding to immobilized VWF and AIM-A1 fragments were detected with horse-radish peroxidase (HRP)-conjugated anti-VHH monoclonal antibody 96A3F5 (Genscript) (1:1000). Purified GPIb-IX complex was coated to the plate using 0.1% Triton X-100, 20 mM Na_2_CO_3_/NaHCO_3_, pH 9.6. Fixed platelets, prepared from human washed platelets followed by two rounds of washing in 4% paraformaldehyde, were coated to the plate using 1% poly-l-lysine (Sigma) in PBS. Bound AIM-A1 fragments were detected with HRP-conjugated anti-HisTag antibody 4E3D10H2/E3 (1:2000). Monoclonal antibody binding to AIM-A1 fragments was detected using HRP-conjugated anti-mouse secondary antibody sc2005 (Santa Cruz Biotechnology) (1:2000). In all cases, plates were washed three times with HEPES buffered saline with 0.1% Tween-20 on a BioTek ELx405 plate washer. After binding and washing, 1-Step Ultra-TMB substrate (ThermoFisher) was added to each well, quenched with 2 M H_2_SO_4_. Absorbance was measured at 450 nm. Empty wells were used to subtract baseline absorbance.

### Construction of TEV-1238–1481

Primers MW002 and MW003 were used to amplify the gene fragment and cloned into the aforementioned modified pcDNA3.1-Hygro vector using BamHI and XhoI sites. The N-terminus of the secreted protein starts with ERHHHHHHHHHHENLYFQGS, followed by VWF residues 1238–1481. Stably transfected Expi293F cells were adapted to SFM4CHO-UT media, following the procedure described above. The target protein was purified by Ni-affinity chromatography and gel filtration as described above for VWF fragments. The VWF fragment was digested with recombinant TEV protease, at a w/w ratio of 11/1, in PBS containing 0.3 mM freshly prepared glutathione and 3 mM oxidized glutathione overnight at 4 °C. The digestion mixture was centrifuged at 4000 *g* at 4 °C and applied onto a His-Trap column, and the flow-through was further purified on a HiLoad 16/600 Superdex 200 pg column. Fractions containing the tag-less AIM-A1 fragment were pooled, concentrated, and mixed with VHH81-6His at a molar ratio of 1/1.5 for 20 min before the AIM-A1/VHH81 complex was separated from unbound VHH81 by gel filtration chromatography (Supplementary Fig. 16D). The purified complex was flash-frozen in liquid nitrogen and stored at −80 °C until use.

### Crystallization, data collection, and structural determination

The AIM-A1/VHH81 complex was concentrated to ~15 mg/ml for crystallization trials using commercial sparse matrix screens (JCSG+, Morpheus, MemGold, Proplex) from Hampton Research (Aliso Viejo, CA) and Molecular Dimensions (Sheffield, UK) in sitting-drop crystallization plates at 10 °C. Single crystals grew from conditions of 3.2 M ammonium sulphate, 0.08 M sodium citrate, pH 5.2 (MemGold, H2). Crystals were harvested and 20% glycerol added as a cryo-protectant, then flash-frozen in liquid nitrogen for the data collection on beamline I04 and Diamond Light Source. Diffraction data were collected from multiple crystals and processed with xia2 and CCP4 suite to 2.1-Å resolution. The structure was then solved using molecular replacement (Phaser) with the A1 domain crystal structure (1AUQ [10.2210/pdb1AUQ/pdb]) and a nanobody homology model without CDR loops as the templates^28,86^. Buccaneer was used to build the initial model followed by manual model building using COOT and refinement with REFMAC. There is evidence of anisotropy in the diffraction data. No TLS refinement was performed. Crystallographic statistics are listed in Supplementary Table 2. The coordinates of the complex structure have been deposited at the Protein Databank (ID: 7A6O [10.2210/pdb7A6O/pdb]). Structures were visualized using PyMOL.

### Parallel-plate flow chamber assay

Parallel-plate flow chamber experiments were performed using a Maastricht Instruments flow chamber (H:50 μm, W:5 mm, L:60 mm). Citrated human whole blood was perfused at room temperature using a Harvard Apparatus Pump 11 Elite. Coverslips were imaged under a Nikon Ti-Eclipse microscope equipped with a 20× Plan Fluor objective lens. Coverslips (22 × 60 mm) were coated with 200 µg/mL bovine type I collagen (Chrono-log) in 5% glucose, pH 2.7 overnight at 37 °C, and blocked with 1% bovine serum albumin in PBS for 1 h. After blood was mixed with 2 µg/mL DIOC-6 (Invitrogen) for 10 min to label platelets and recalcified with 5 mM CaCl_2_, 1 μM ARC1172, 880 nM or 2.64 μM VHH81-6His, or 880 nM VHH81-6His and 15 µg/mL 11A8 was added and mixed for 10 min before perfusion. After the coverslip was assembled into the flow chamber, it was washed with modified Tyrode’s buffer with 5 mM glucose for 1 min. Blood was perfused for 4 min at each shear rate. The chamber was subsequently washed with modified Tyrode’s buffer for 2 min at the same shear rate and immediately imaged in the FITC filter (excitation 480 nm/30 nm, barrier 535 nm/45 nm). On average 20 fluorescent images were collected, with collagen deposited on slides often visible in bright-field images. Area covered by adhered platelets was calculated using FIJI, thresholding for each image using the greyscale LUT. Experiments at various shear rates were performed using blood from 2 to 3 donors with similar results observed throughout.

### Statistical analysis

Data are shown as mean ± standard deviation, with a number of replicates indicated in relevant figure legends. Where applicable, one or two-way ANOVA with Tukey’s multiple comparison correction was performed to analyze platelet coverage as indicated in the figure legends. For BLI experiments, all binding interactions were fit to a heterogeneous ligand binding model in the Data Analysis HT software from ForteBio. Steady-state analysis was performed in Prism by fitting concentration-response curves to a hyperbola. Relevant fitting parameters for force spectroscopy are described above.

### Reporting summary

Further information on research design is available in the Nature Research Reporting Summary linked to this article.

## Supplementary information

Supplementary Information

Description of Additional Supplementary Files

Supplementary Video 1

Supplementary Video 2

Reporting Summary

## Data Availability

Data supporting the findings of this manuscript are available from the corresponding authors upon reasonable request. A reporting summary for this Article is available as a Supplementary Information file. Source data are provided with this paper. Protein coordinates and structure factors have been deposited in the RCSB Protein Data Bank under code 7A6O. Source data are provided with this paper.

## Source data

Source data
