# Supplementary material for: Activation of von Willebrand factor via mechanical unfolding of its discontinuous autoinhibitory module
Source: Nat Commun. 2021 Apr 21;12:2360. doi: 10.1038/s41467-021-22634-x (PMC8060278; doi:10.1038/s41467-021-22634-x)
Supplement: Supplementary file 1 — Supplementary Information [file 41467_2021_22634_MOESM1_ESM.pdf]

Supplemental Information for

Activation of von Willebrand factor via mechanical unfolding of its discontinuous autoinhibitory module

Nicholas A. Arce, Wenpeng Cao, Alexander K. Brown, Emily R. Legan, Moriah S. Wilson, Emma-Ruoqi Xu, Michael C. Berndt, Jonas Emsley, X. Frank Zhang, and Renhao Li

Supplemental Tables

Supplementary Table 1. Data collection and refinement statistics of solved crystal structure of a recombinant VWF fragment (residues 1238-1481) in complex with VHH81.

**Space group**	P 4_32_12
**Cell dimensions**	
a, b, c (Å)	65.2, 65.2, 233.3
α, β, γ (°)	90, 90, 90
X-ray Source	DLS I04
Wavelength (Å)	0.9795Å
Data Range (Å)	62.8 – 2.1
Reflections unique	19344
R_{merge} [a]	0.048 (0.850)
I / σI	15.1 (1.7)
Completeness (%)	92.4 (70.9)
Multiplicity	5.2 (4.9)
**Refinement**	
Resolution (Å)	62.8 – 2.1
R_{work}[b] / R_{free} [c]	0.191/0.251 (0.36/0.64)
Half-set correlation CC(1/2)	0.999 (0.645)
**Atoms**	
Nonhydrogen protein atoms	2626
Solvent	160
Ion (SO_4)	5
B-factors average (Å^2)	54.0
**R.M.S.D**	
Bond Lengths (Å)	0.0097
Bond angles (°)	1.727
**Validation**	
MolProbity Score	2.16 (70th percentile[d])
Clashscore, all atoms	5.88 (97th percentile[d])
**Ramachandran Plot**	
Most favoured	317 (96.35%)
Allowed	8 (2.43%)
Outliers	4 (1.22%)

[a] *R*merge = ∑hkl∑j |Ihkl,j - Ihkl|/∑hkl∑jIhkl,j, where Ihkl is the average of symmetry-related observations of a unique reflection. In brackets on the right are values from the outer, high resolution shell of the diffraction data.
[b] *R*work = ∑hkl ||*F*obs(hkl)|-|*F*calc(hkl)||/∑hkl|*F*obs(hkl)|.
[c] *R*free= The cross-validation *R* factor for 5% of reflections against which the model was not refined.
[d]100th percentile is the best among structures of comparable resolution.

Supplementary Table 2. Primers used in this study

Primer Name	5' Sequence 3'
IgK-1268F	GCTAGCATGGAGACAGACACACTCCTGCTATGGGTGCTGCTGCTCTGGGTTCCAGGTTCCACTGGTAAGCACGATTTCTACTGCAGCAGG
1268XbaStopR	TCTAGATCAATGATGATGATGATGATGATGATGATGATG
1493F	CTAGATACTAGTCAGGAGCCGGGA
1493RSpyStop	TAGCCTCTCGAGTCACTTAGTCGGCTTATATGCATCGACCATAACGATATGGGCATGATGATGATGATGATGATGATGATGATG
EL007	CGGAACCGCCGTTGGACGATTTCTACTGC
EL008	GCAGTAGAAATCGTCCAACGGCGGTTCCG
EL003	GCGACCGTCAGAGCTGCAGCGCATTGCCAGCCAG
EL004	GGCTGGCAATGCGCTGCAGCTCTGACGG
GPIba_Biotag	AGCTGGATCCGGCCTGAACGACATCTTCGAGGCTCAGAAAATCGAATGGCACGAACACCCCATCTGTGAGGTCTCC
GPIba290_2xFLAGstop	CTGACTCGAGTCATTTGTCATCATCATCCTTATAGTCTTTGTCATCATCATCCTTATAGTCACGCACCTTATCGCCCTCAGT
pD14_VHH81F	GCATCTCGAGTCAGTGGTGGTGATGATGATGGGATGACACAGTTACC
pD14_VHH81R	GCAGCATATGGAGGTTCAACTTGTGGAGAG
MW002	AATCAGGATCCCAGGAGCCGGGAGGCCTG
MW003	TAAGACTCGAGTTACCCCGGGCCCACAGTGAC

Supplemental Figures

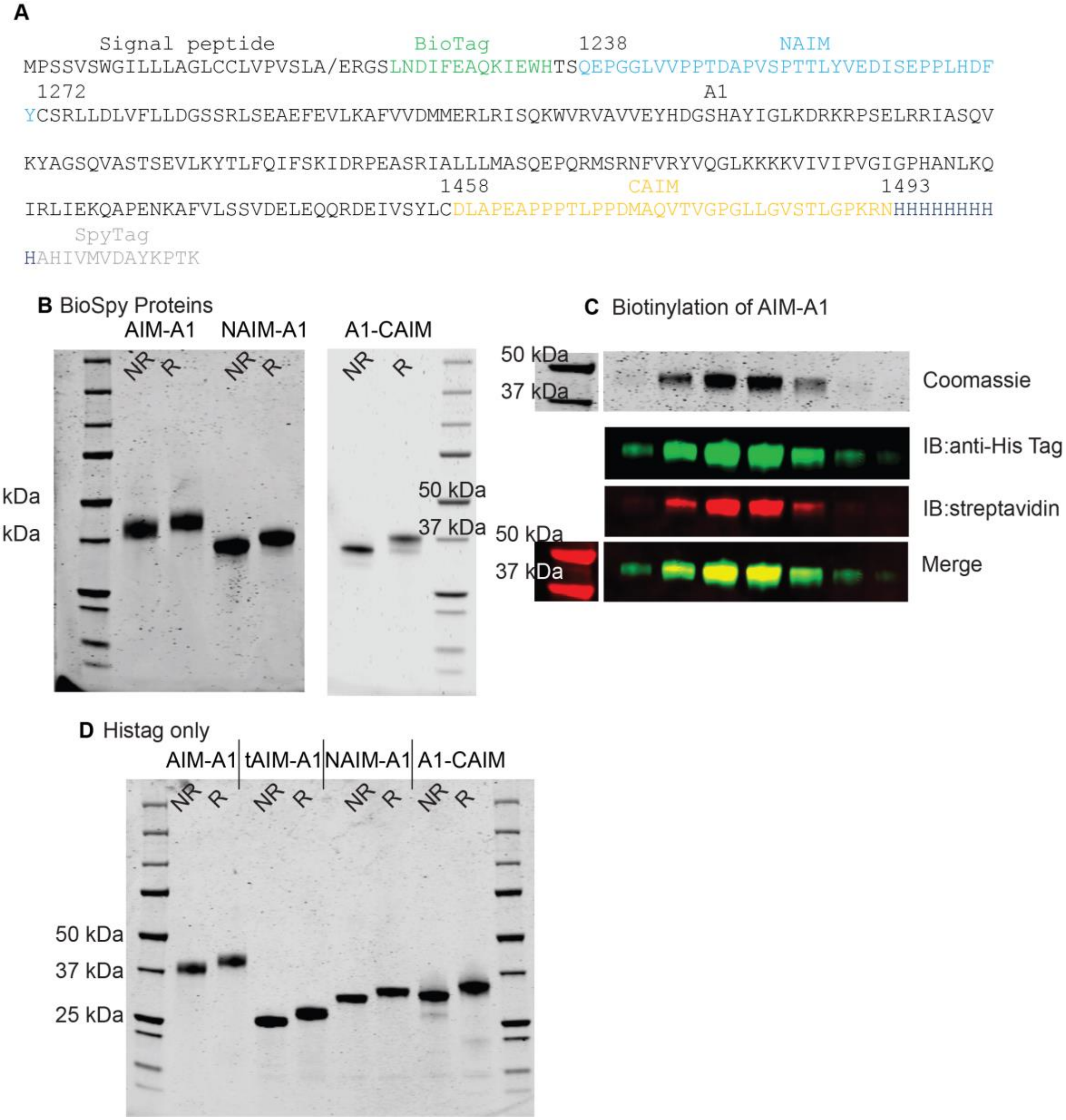

Supplementary Figure 1. Sequence of Biospy AIM-A1 and expression of rVWF Biospy fragments, rVWF-His Fragments, and GPIbα LBD.
(A) Sequence of Biospy 1238-1493 with corresponding labels for NAIM (cyan), A1 (black), CAIM (orange), Biotag (green), and SpyTag (grey). (B) Coomassie stained gels of Biospy proteins used for single-molecule experiments. The first lane corresponds to the molecular weight markers, with 50 kDa and 37 kDa labelled. These gels are the result after 4 successful protein purification run. (C) Representative size exclusion fractions of Biospy 1238-1493 (AIM-A1) stained with Coomassie blue (top), blotted for anti-His tag (middle), and streptavidin (bottom). Merged images results in a yellow color, indicating that the protein bands are reactive to both streptavidin and anti-His tag antibody. These gels are the result after 2 successful protein purification run following ~3 runs to optimize preparation conditions. (D) Coomassie stained

gels of rVWF fragments bearing only a poly-histidine tag on the C-terminus used for binding experiments and platelet aggregation. The gel shows the purity of various proteins that are prepared from 3-5 successful protein purification runs.

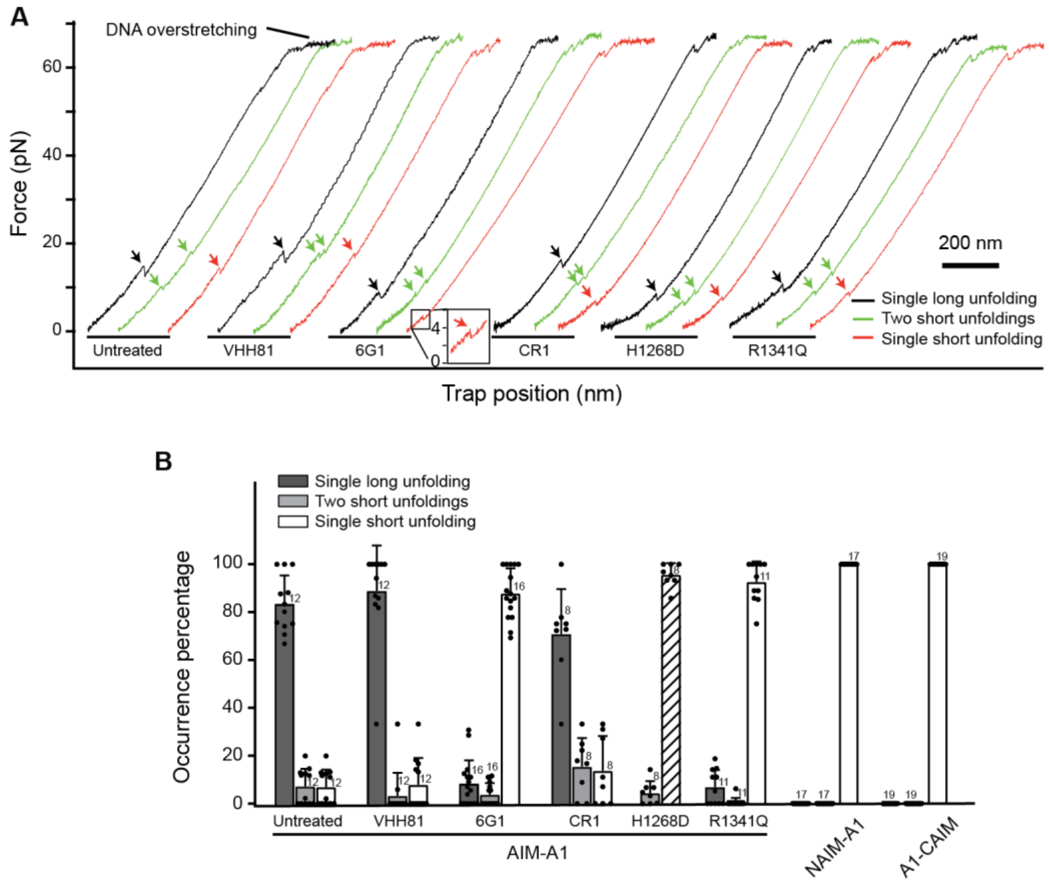

Supplementary Figure 2. Force-extension traces and unfolding extension grouping for all constructs tested.

(A) Representative force-extension traces (generated by pulling at 200 nm/s) of AIM-A1 alone, and treated with VHH81, CR1 or 6G1. Force extension traces of the type 2B VWD mutants are also included. The extension event(s) in each trace is marked by an arrowhead. Single unfolding events are represented by black traces, with two short unfolding traces in green and one short unfolding in red. Inset: a zoom-in view of a low force unfolding event. (B) Percentage of three unfolding types for different constructs pulled at 200 nm/s after relaxation under 1 pN for 1 second. The AIM-A1 construct unfolds as a singlet unfolding the majority of the time, while NAIM-A1 and A1-CAIM have lower force singlet unfolding. In some instances, two lower force unfolding events can occur, corresponding to NAIM and CAIM unfolding separately. *Mutation H1268D resulted in a shortened AIM unfolding extension. As a result, we were not able to distinguish single-short and single-long unfolding signals. All single unfolding events are represented by the stripe-patterned bar. Error bars represent standard deviation. The data was obtained from n=12, 12, 16, 8, 8, 11, 17 and 19 biologically independent single-molecule tethers for untreated, VHH81, 6G1, CR1, H1268D, R1341Q, NAIM-A1, and A1-CAIM, respectively.

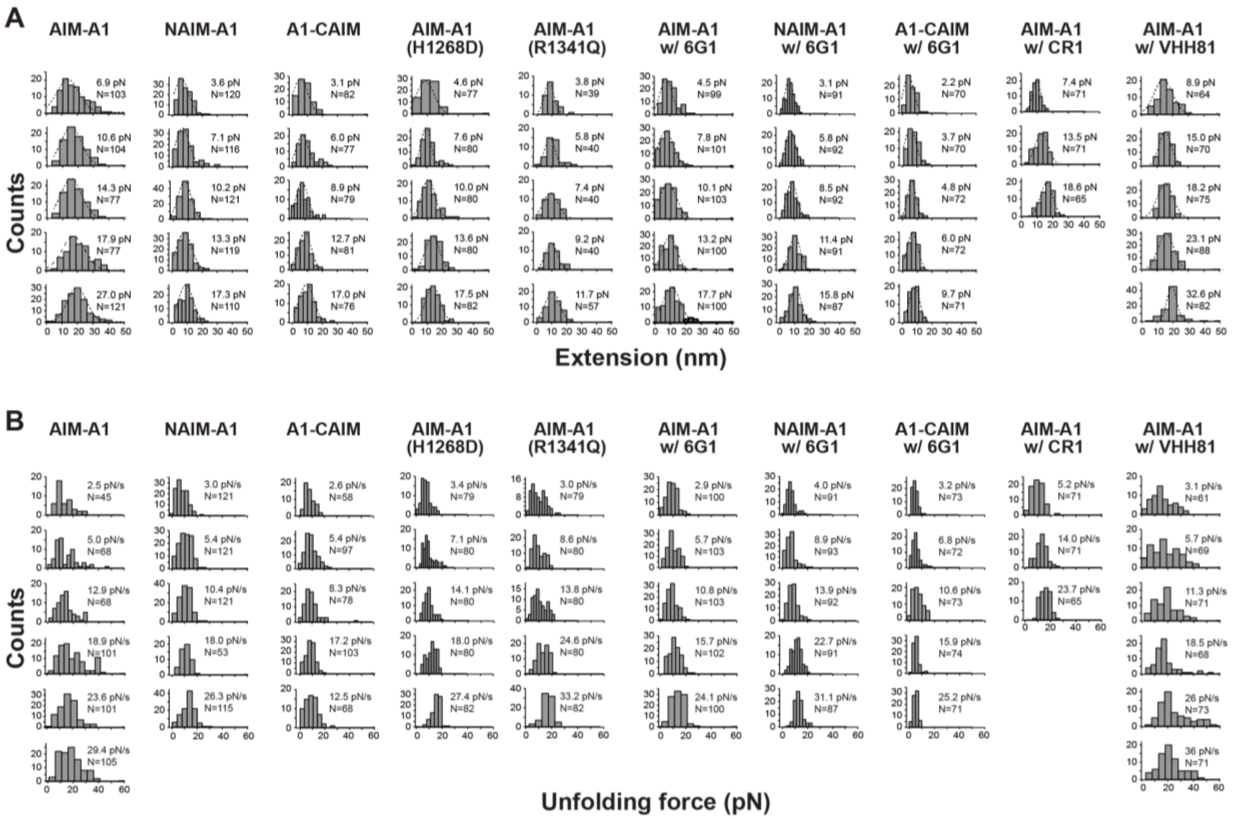

Supplementary Figure 3. Histograms of AIM unfolding in various fragments and additives. (A) Unfolding extension histograms at various forces. A Gaussian fit is overlaid as a dashed line on each histogram. (B) Unfolding force histograms at various loading rates.

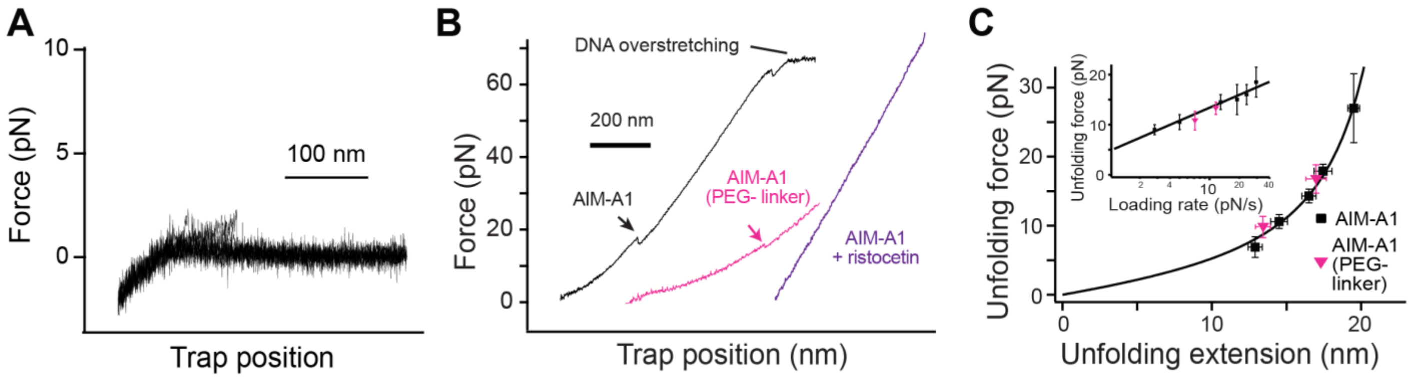

Supplementary Figure 4. The DNA handle does not interfere with AIM unfolding.
(A) Overlay of eight representative consecutive retraction traces of a DNA handle-coupled bead interacting with an AIM-A1 coupled bead. To address a potential concern that DNA may bind A1 through electrostatic interactions, AIM-A1 was immobilized on one bead and the DNA handle alone (no SpyCatcher) on another bead, no specific binding forces were observed between AIM-A1 and DNA, as non-specific forces between two beads are typically below 2 pN. (B) Typical force-trap position curves generated by pulling (200 nm/s) AIM-A1 using DNA handle method (black) and using PEG-linker (magenta), and AIM-A1 using DNA handle in the presence of 1 mg/ml ristocetin (purple). (C) Relationship between unfolding force and extension for unfolding events in AIM-A1 using DNA handle method (black) and using PEG-linker (magenta). Force data are presented as mean values ± standard deviation., and extension data are presented as the peak of the Gaussian fit ± the FWHM of Gaussian fit divided by the square root of counts. The data are fitted to a worm-like chain model (solid lines). Inset: the relationship between the most probable unfolding forces plotted against loading rate. Unfolding force data are presented as the center of the tallest bin of the histogram. Error bars are one-half of the bin width for unfolding force. The solid lines are fits to the Bell-Evans model. Note that although there was no overstretching signal in PEG-linked beads, similar magnitudes of AIM unfolding forces and extensions were observed. The data was obtained from n=52 and 16 biologically independent single-molecule tethers for AIM-A1 and PEG, respectively.

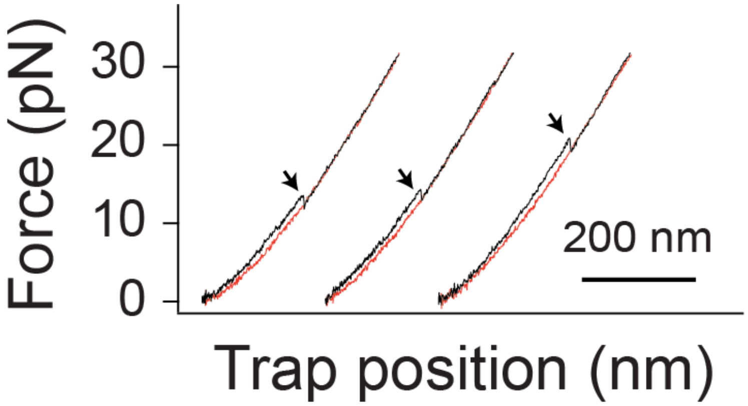

Supplementary Figure 5. Representative unfolding-refolding traces (generated by pulling at 200 nm/s) of AIM-A1. Pulling and relaxation traces are shown in black and red, respectively. Arrows indicate unfolding (black) events.

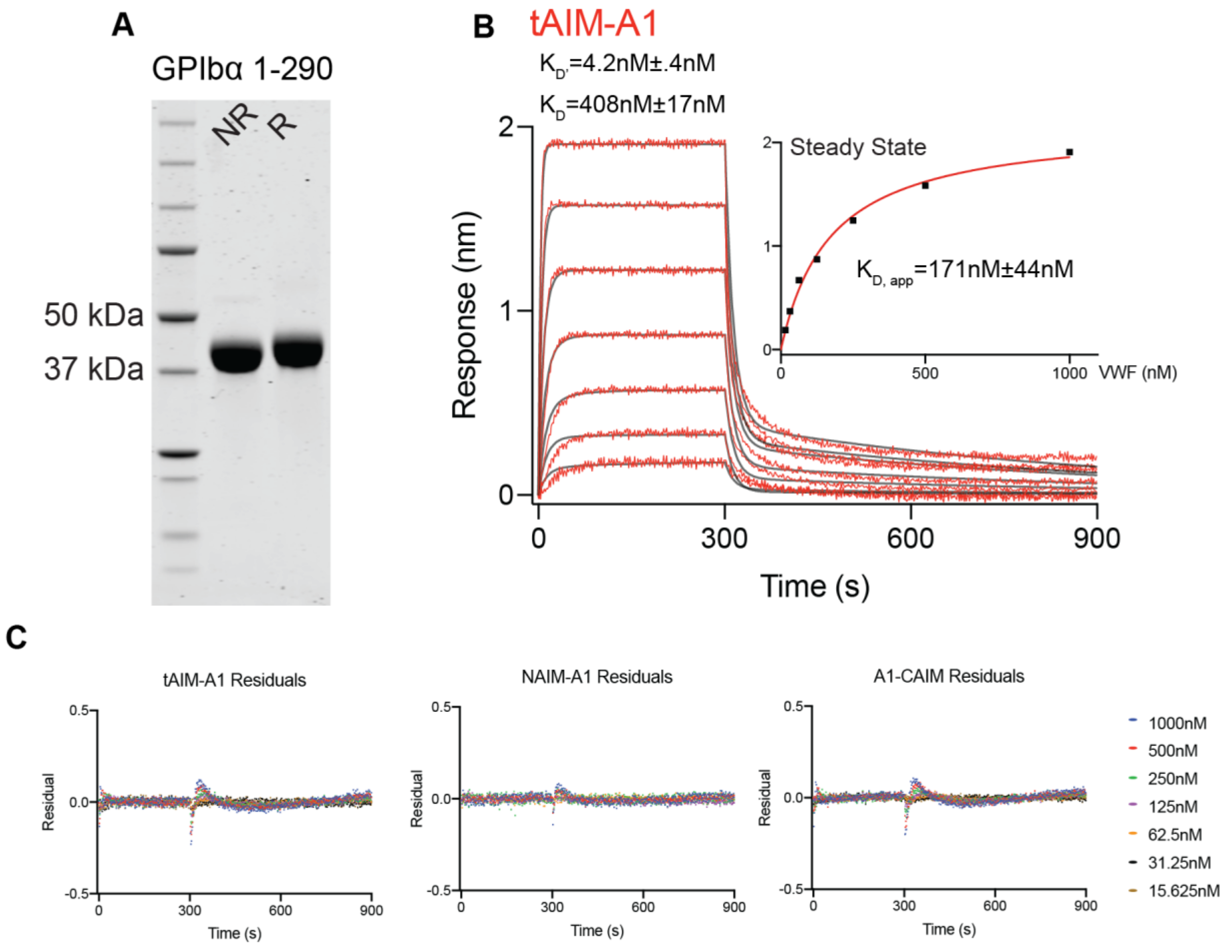

Supplementary Figure 6. Truncation of the AIM yields a highly active A1 fragment towards GPIbα.

(A) Coomassie blue stained gel of recombinant GPIbα LBD under non-reducing and reducing conditions. Lane 1 are molecular weight markers with 37 kDa and 50 kDa indicated. The gel shows one of the two batches of equally purified recombinant GPIbα LBD. (B) Sensorgram of rVWF 1261-1472 (tAIM-A1) binding to immobilized GPIbα. Fitting traces are underlaid in black. Inset is steady state fit to a hyperbola. K_D and $K_{D'}$ are presented as mean ± standard devation of individual fits while $K_{D,app}$ is shown as mean ± 95% confidence interval. (C) Residual plot versus time for the fittings subtracted from observed sensorgrams for GPIbα to tAIM-A1, NAIM-A1 and A1-CAIM.

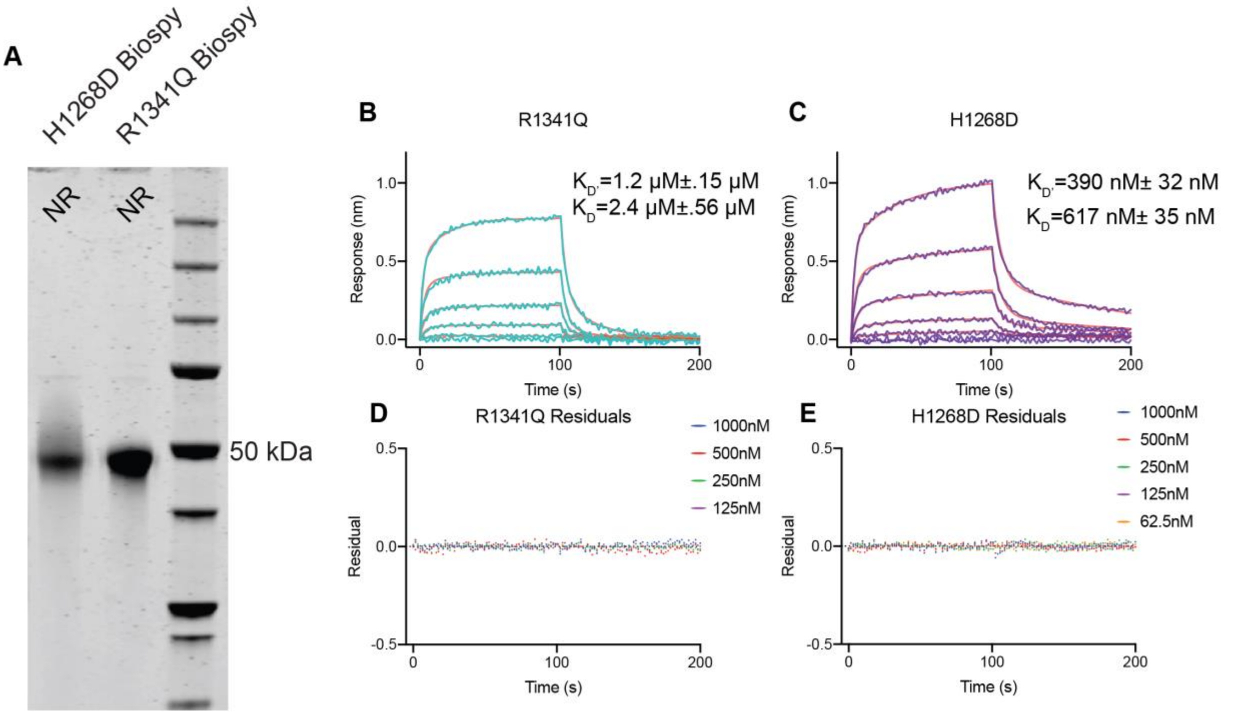

Supplementary Figure 7. Expression and binding of Type 2B VWD AIM-A1 mutants. (A) Coomassie stained gel of H1268D and R1341Q Biospy mutants under non-reducing conditions. The lane on the right shows molecular weight markers with that of 50 kDa marked. The gel shows the result after two successful purification runs of H1268D and 3 runs of R1341Q. (B, C) Sensorgrams of a dilution series from 1 μM to 15.6 nM H1268D and R1341Q binding to immobilized GPIbα. Fitting overlaid in red is a heterogenous ligand binding model, K_D and $K_{D'}$ are presented as mean ± standard deviation of global fits for concentrations showing observable binding responses. (D, E) Fitting residual plots versus time for observed sensorgrams.

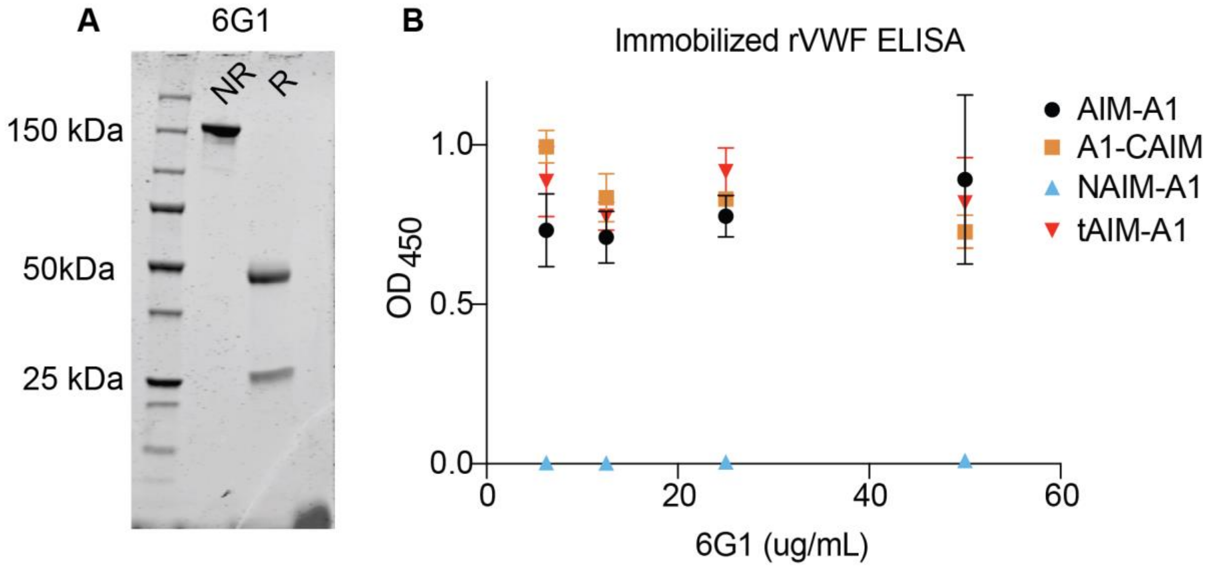

Supplementary Figure 8. Expression and binding site verification of monoclonal antibody 6G1. (A) Coomassie stained gel of purified 6G1. Molecular weight markers are run in lane 1, with 150, 50 and 25 kDa highlighted. This gel is the result of 1 successful protein purification run that produced 4 mg of 6G1. (B) rVWF fragments were coated to an ELISA plate, and a dilution series of 6G1 binding was detected with anti-mouse secondary antibody. Data shown are means ± standard deviation, where each condition is done in triplicate (n=3). 6G1 binds to all recombinant fragments, except 1238-1461, confirming the binding site to previous works.

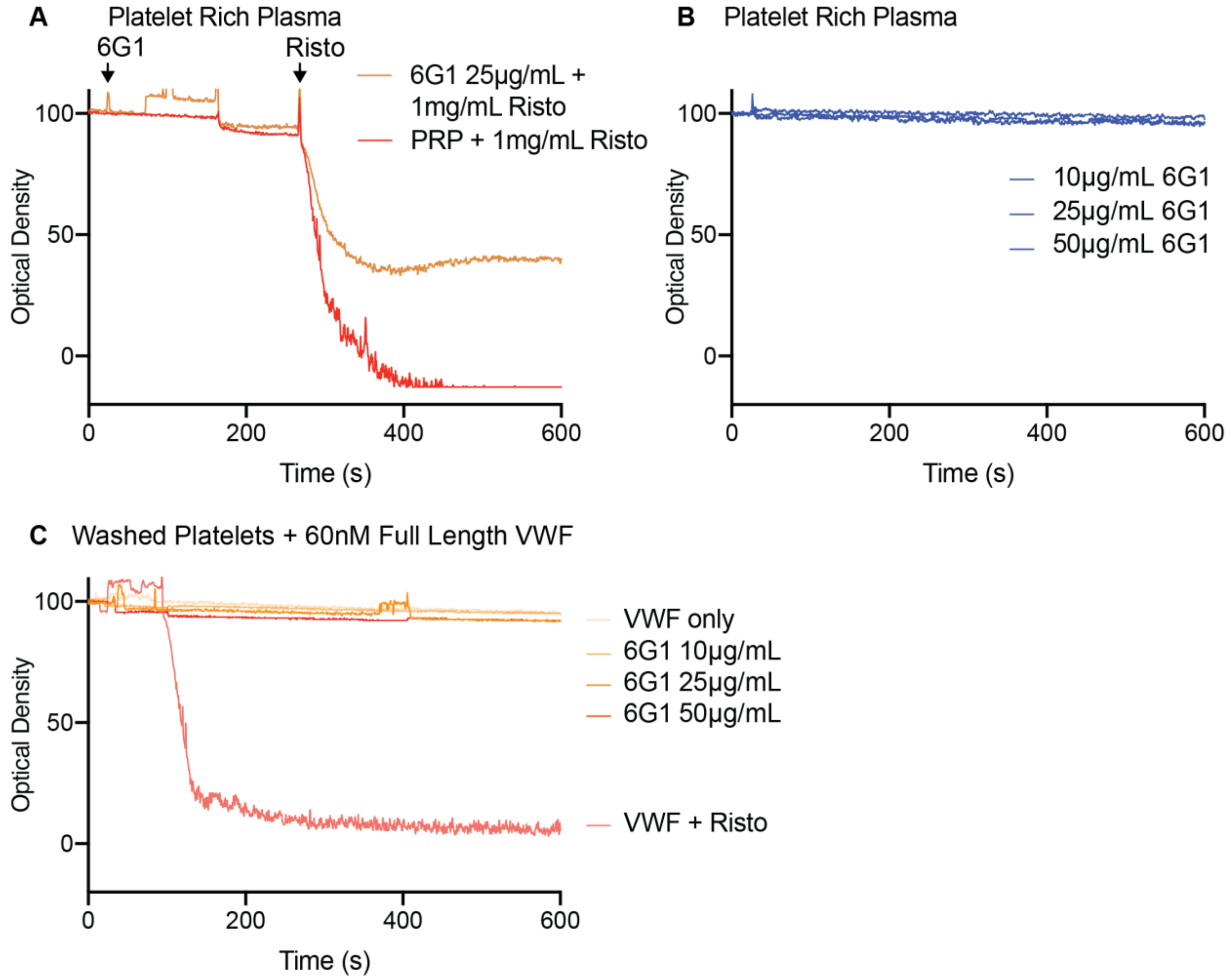

Supplementary Figure 9. The effects of 6G1 on platelet aggregation in the presence of full-length VWF.
Aggregometry traces of 6G1 in PRP (A), the effect of 6G1 on ristocetin induced platelet aggregation (B) and aggregometry of washed platelets with 60 nM full length VWF with 6G1 or ristocetin (C). 6G1 is able to reduce ristocetin induced platelet aggregation in platelet rich plasma. 6G1 is unable to induce aggregation of full length VWF in washed platelets or in platelet rich plasma.

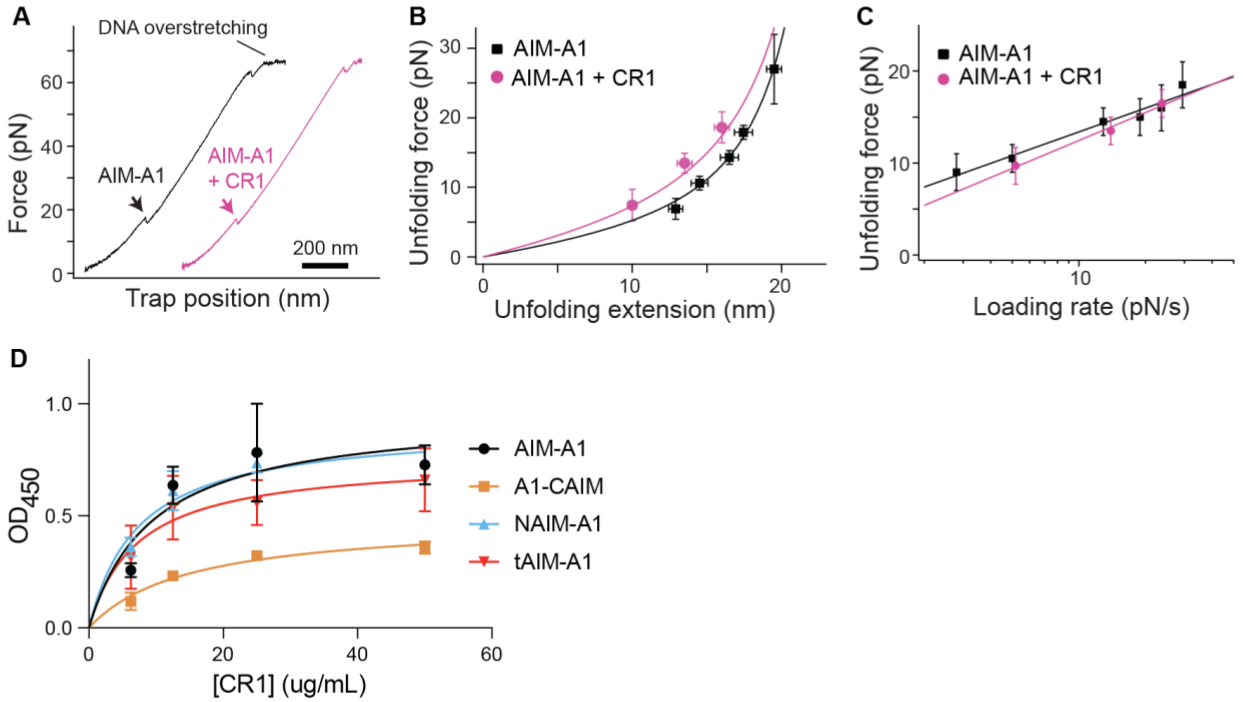

Supplementary Figure 10. Single-molecule characterization of AIM-A1 unfolding in the presence of monoclonal antibody CR1.

(A) Force-extension unfolding traces of AIM-A1 in the presence of CR1. The extension event in each trace is marked by an arrowhead. (B) Superimposed plots of unfolding force versus unfolding extension data and their fits to the worm-like chain model. Force data are presented as mean values ± standard deviation., and extension data are presented as the peak of the Gaussian fit ± the FWHM of Gaussian fit divided by the square root of counts. The data was obtained from n=52 and 28 biologically independent single-molecule tethers for AIM-A1 and CR1, respectively. (C) Regression of most probable unfolding forces fit to the Bell-Evans model. Unfolding force data are presented as the center of the tallest bin of the histogram . Error bars are one-half of the bin width for unfolding force. The data was obtained from n=52 and 28 biologically independent single-molecule tethers for AIM-A1 and CR1, respectively. (D) ELISA of immobilized VWF fragments to a dilution series of CR1. Data shown are means ± standard deviation, where each condition is done in triplicate (n=3). Data are fit to a hyperbola. CR1 binds to all VWF fragments tested.

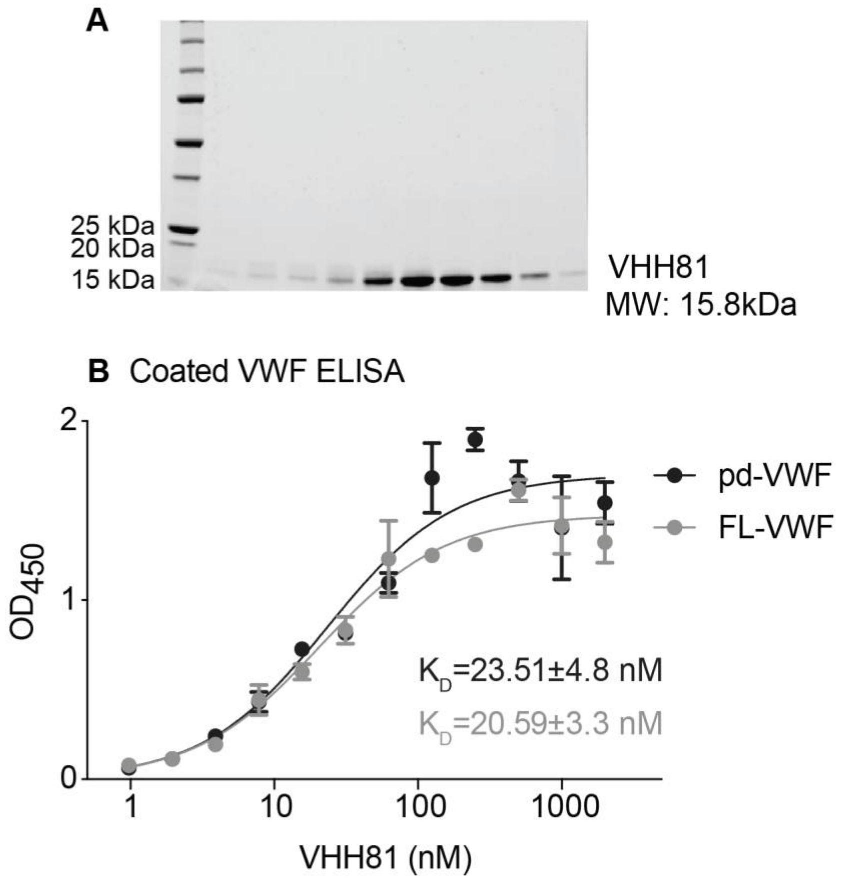

Supplementary Figure 11. Expression and binding of VHH81 to multimeric VWF.
(A) Representative Coomassie stained gel of size exclusion fractions of VHH81. Expected MW is 15.8 kDa. Molecular weight markers are in lane 1 with weights of 25, 20, and 15 kDa explicitly labelled. The gel shows one of the 5 batches of equally purified VHH81. (B) Binding isotherms of VHH81 to immobilized purified VWF (grey) and plasma derived VWF in Humate-P (black). Data shown are means ± standard deviation, where each condition is done in quadruplicate. K_D is presented as mean ± standard deviation from hyperbolic fitting.

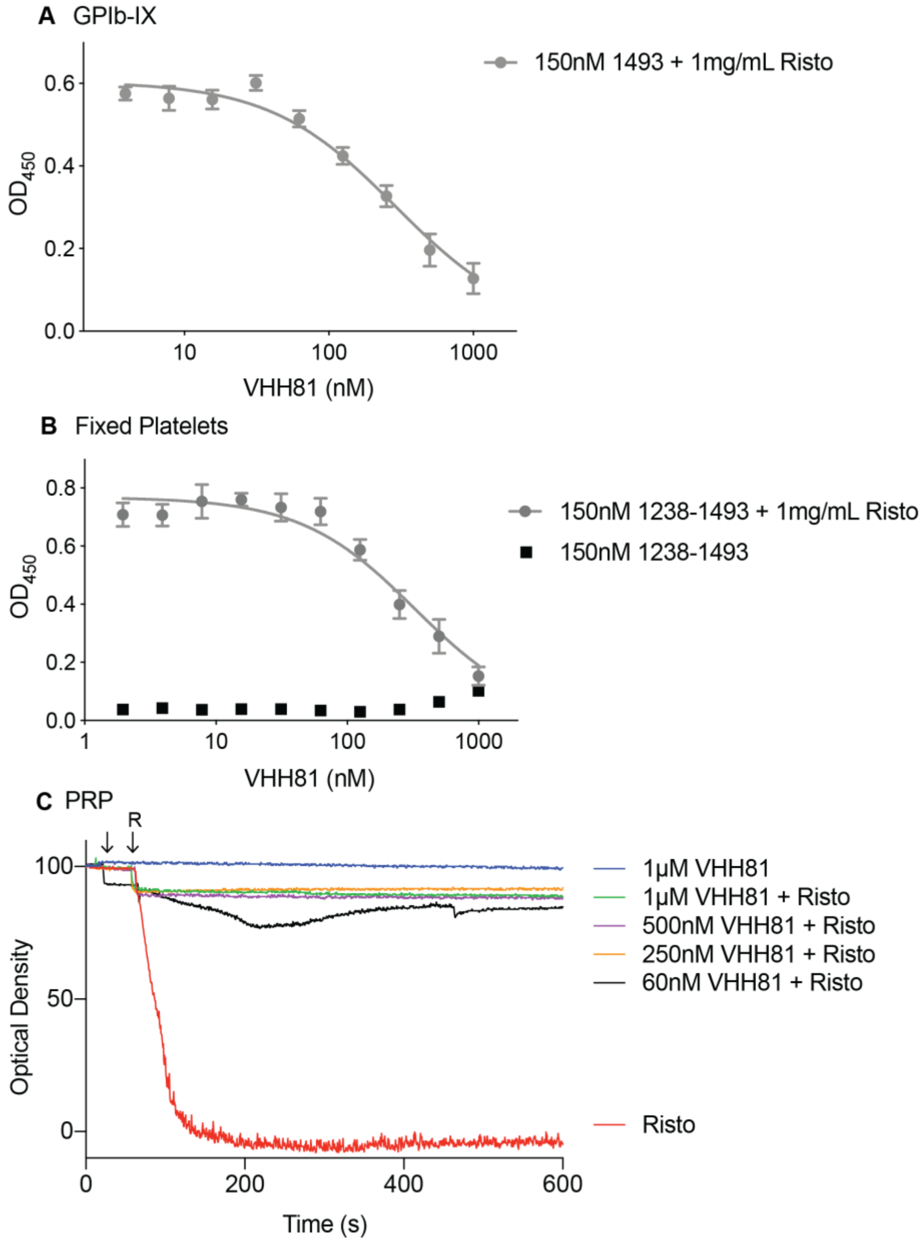

Supplementary Figure 12. VHH81 inhibition of VWF binding to GPIbα.
(A) 150 nM AIM-A1 + 1 mg/mL ristocetin was incubated with a titration of VHH81 to inhibit binding to purified GPIb-IX complex. Data shown are means ± standard deviation, where each condition is done in triplicate. (B) 150 nM AIM-A1 + 1 mg/mL ristocetin was incubated with a titration with VHH81 (grey) and without (black) to inhibit binding to fixed platelets. Data shown are means ± standard deviation, where each condition is done in triplicate. (C) Aggregation traces of platelet-rich plasma with various amounts of VHH81 in response to 1 mg/mL ristocetin. Arrows indicate the addition of VHH81 followed by the addition of 1 mg/mL ristocetin.

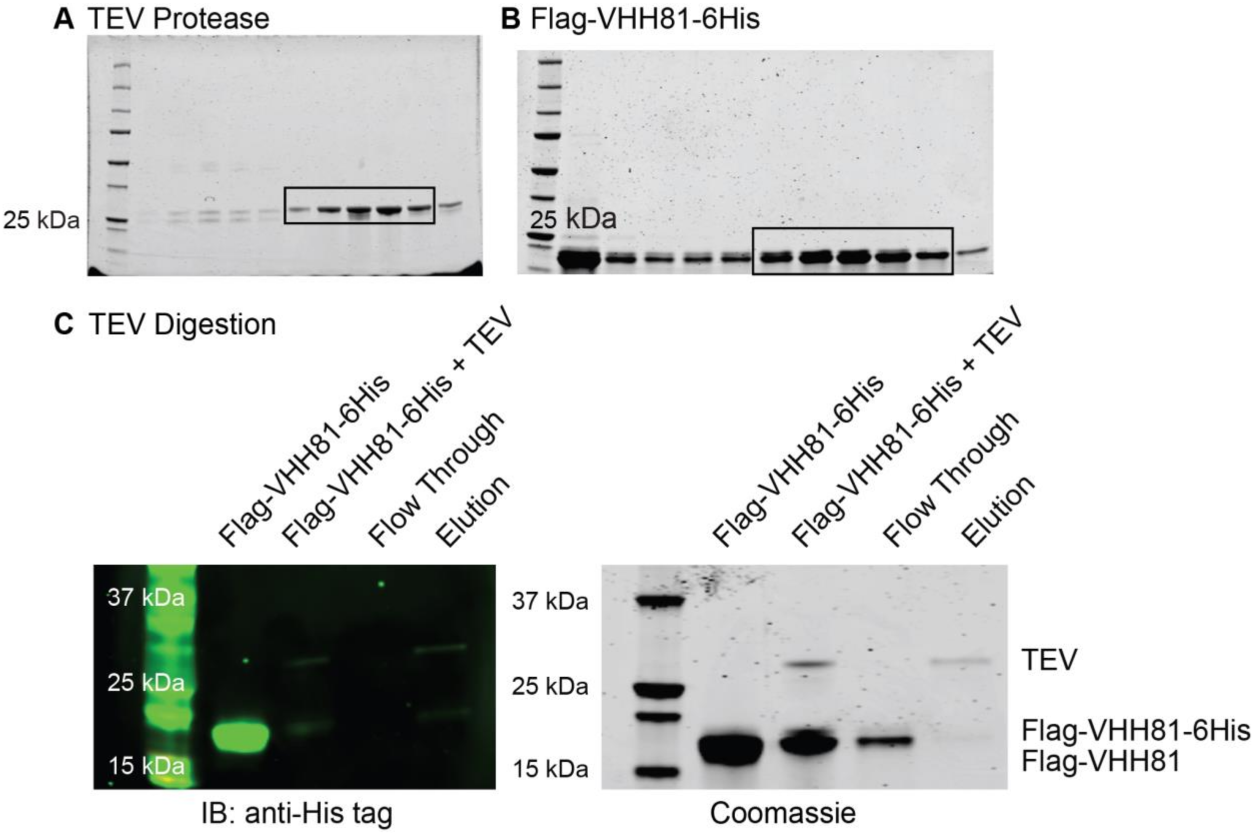

Supplementary Figure 13. Expression and purification of Flag-VHH81-6His.
(A) Size exclusion fractions of recombinant TEV protease. (B) Size exclusion fractions of Flag-VHH81-6xHis, bearing an N-terminal FLAG tag and C terminal TEV cleavable 6xHis Tag. Molecular weight markers are in the first lanes, with 25 kDa highlighted. (C) Immunoblot verification of Histag removal of in flow through of a Ni-sepharose column. TEV treatment of the protein removes identification of the protein by western blot through anti-His tag antibody. TEV-protease contains a His tag that was caught in the Ni-column. These gels demonstrate the quality of Flag-VHH81-6His from 2 successful purification runs, which followed 4 cycles of condition optimization.

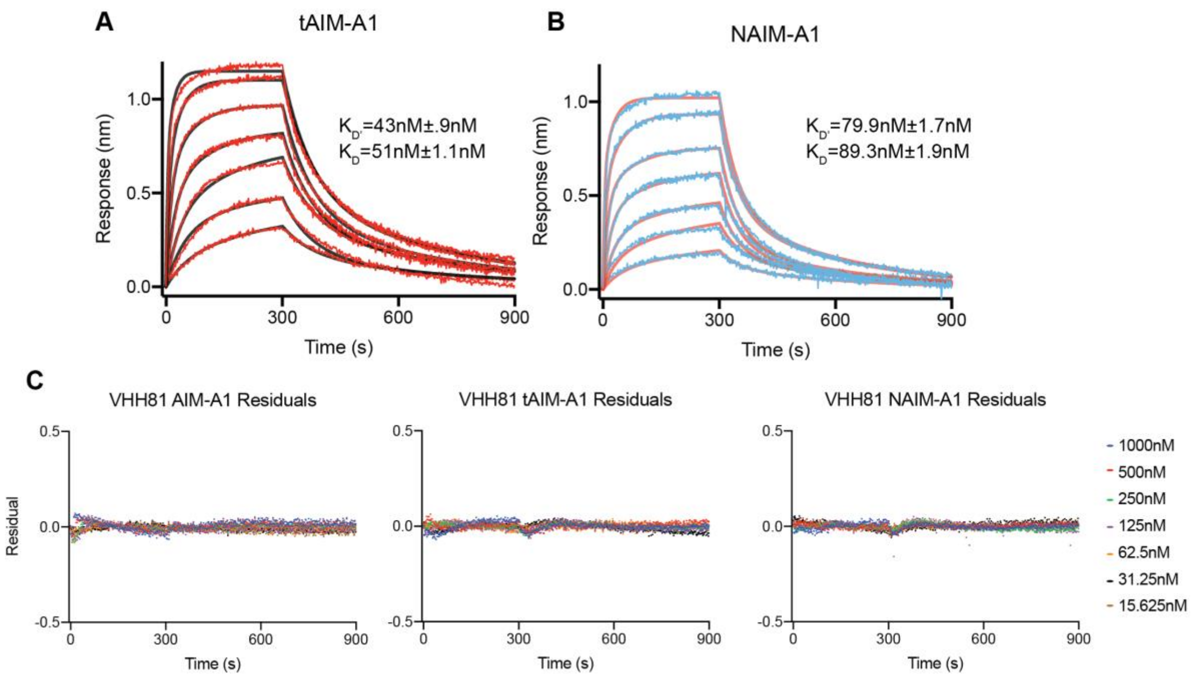

Supplementary Figure 14. Binding of Flag-VHH81 to rVWF fragments containing parts of the NAIM measured by BLI.

(A, B) Binding sensorgrams of tAIM-A1 (red) and NAIM-A1 (cyan) to Flag-VHH81. Global fit is overlaid in black in A, and red in B. K_D and $K_{D'}$ are presented as mean ± standard deviation of global fits. (C) Residual plot versus time for the fittings subtracted from observed sensorgrams.

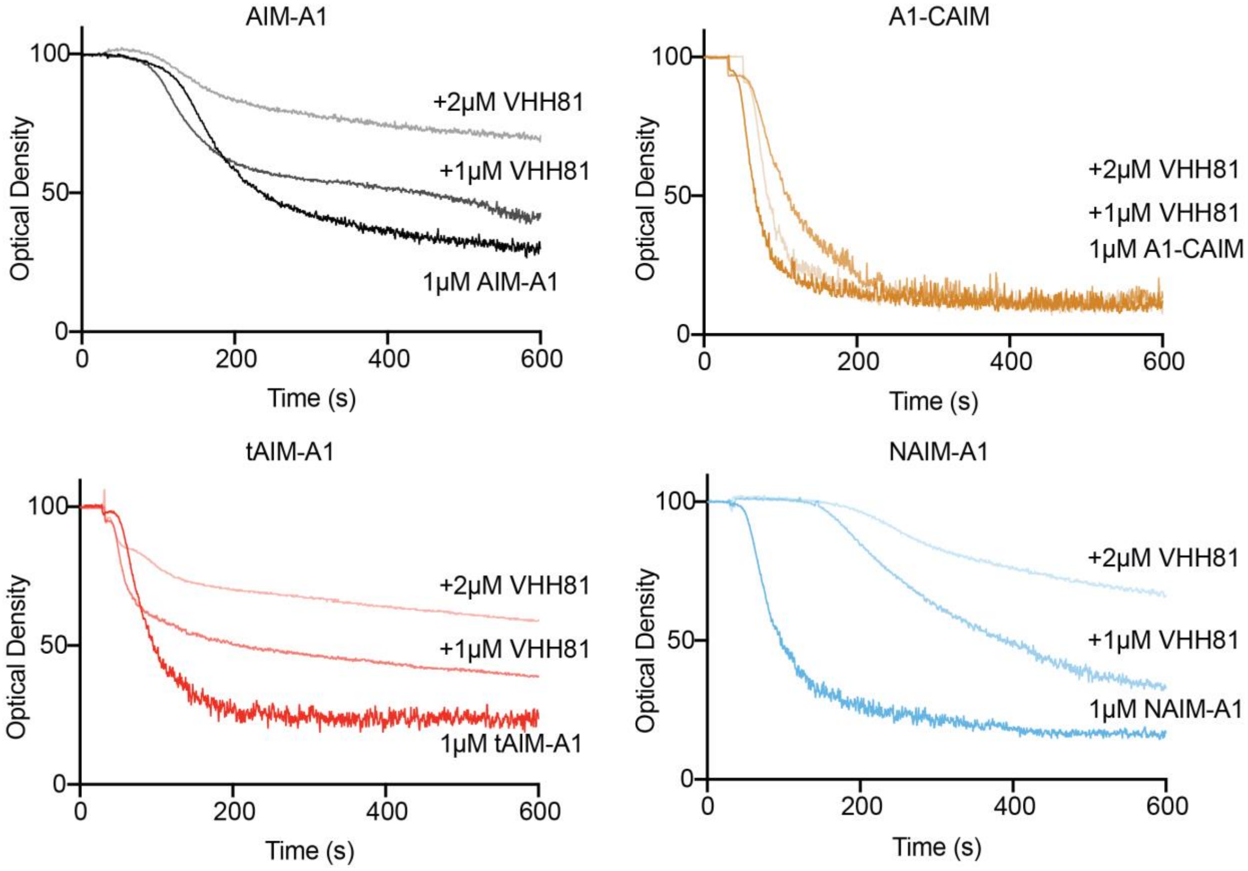

Supplementary Figure 15. VHH81 inhibition of rVWF fragment induced washed platelet aggregation. Representative washed platelet aggregometry inhibition by 0,1, or 2 μM VHH81 of various AIM-A1 proteins (1μM).

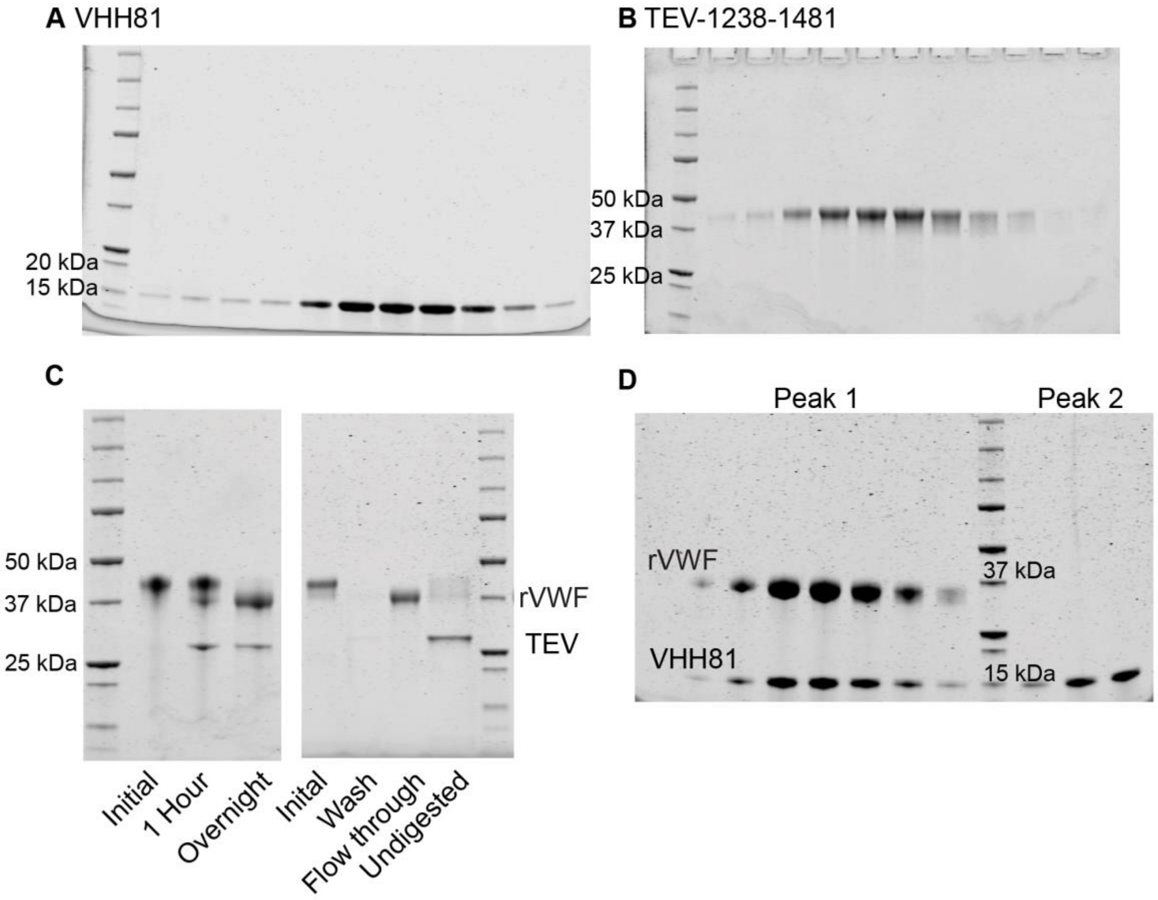

Supplementary Figure 16. Expression and purification of rVWF-VHH81 complex used for crystallization.

Size exclusion fractions of VHH81 (A) and TEV-1238-1481 (B). (C) Cleavage of TEV-1238-1481. (D) Coomassie stained SDS-PAGE gel of size exclusion fractions of the formed complex. Peak 1 was used for crystallization trials. These gels demonstrate the quality of the protein following a protein purification run that produced ~20 mg of purified protein complex. Similar results were obtained during 3 successive cycles of condition optimization.

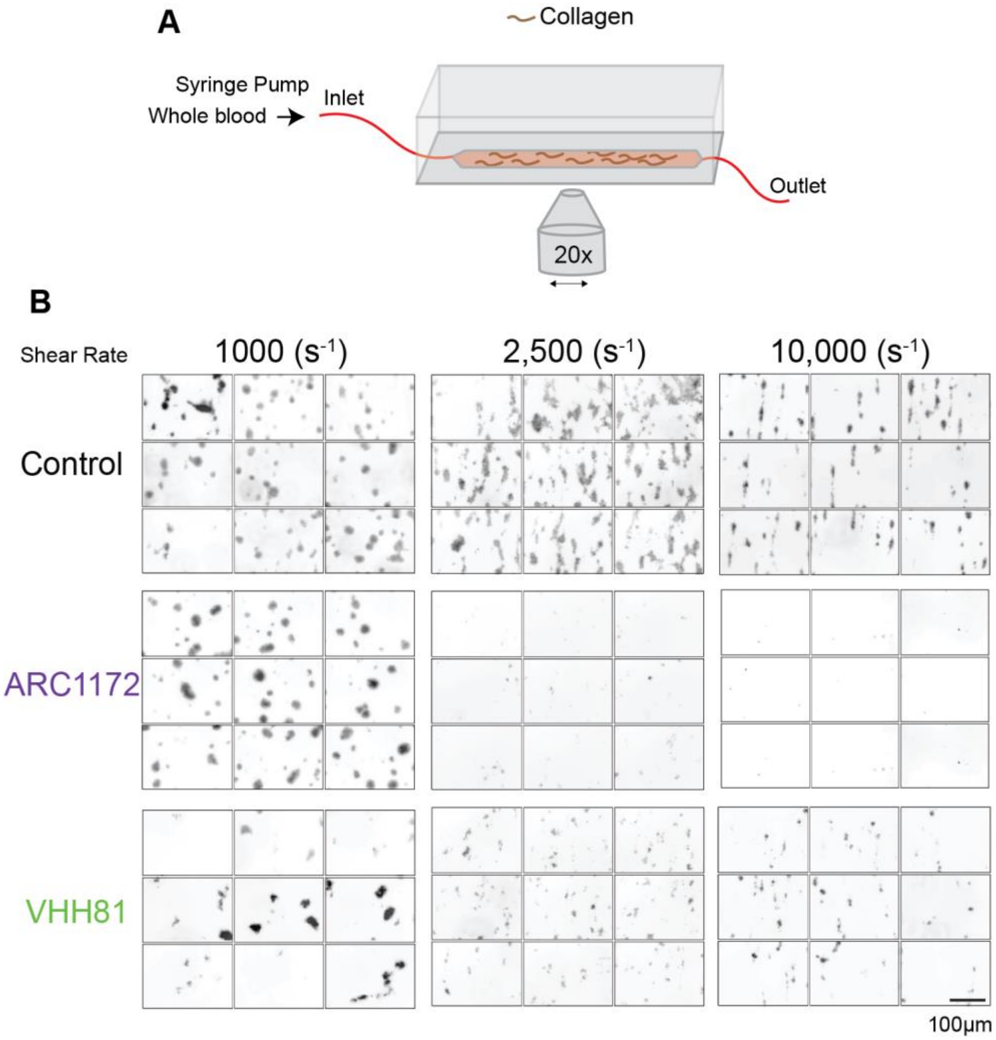

Supplementary Figure 17. Parallel-plate flow chamber set up and representative images of whole blood platelet adhesion to a collagen substrate in the presence of VHH81 or ARC1172. (A) Cartoon of the flow chamber set-up. Labelled whole blood is perfused into the chamber at desired shear rates and imaged immediately after 4 minutes of perfusion followed by 2 minutes of washing with modified Tyrode's buffer for 2 minutes. (B) Representative flow chamber images of platelet adhesion on collagen coated substrate. Images were collected on the FITC channel as platelets were labelled with DIOC-6, and a greyscale LUT as applied in FIJI. Scale bar, 100 µm.

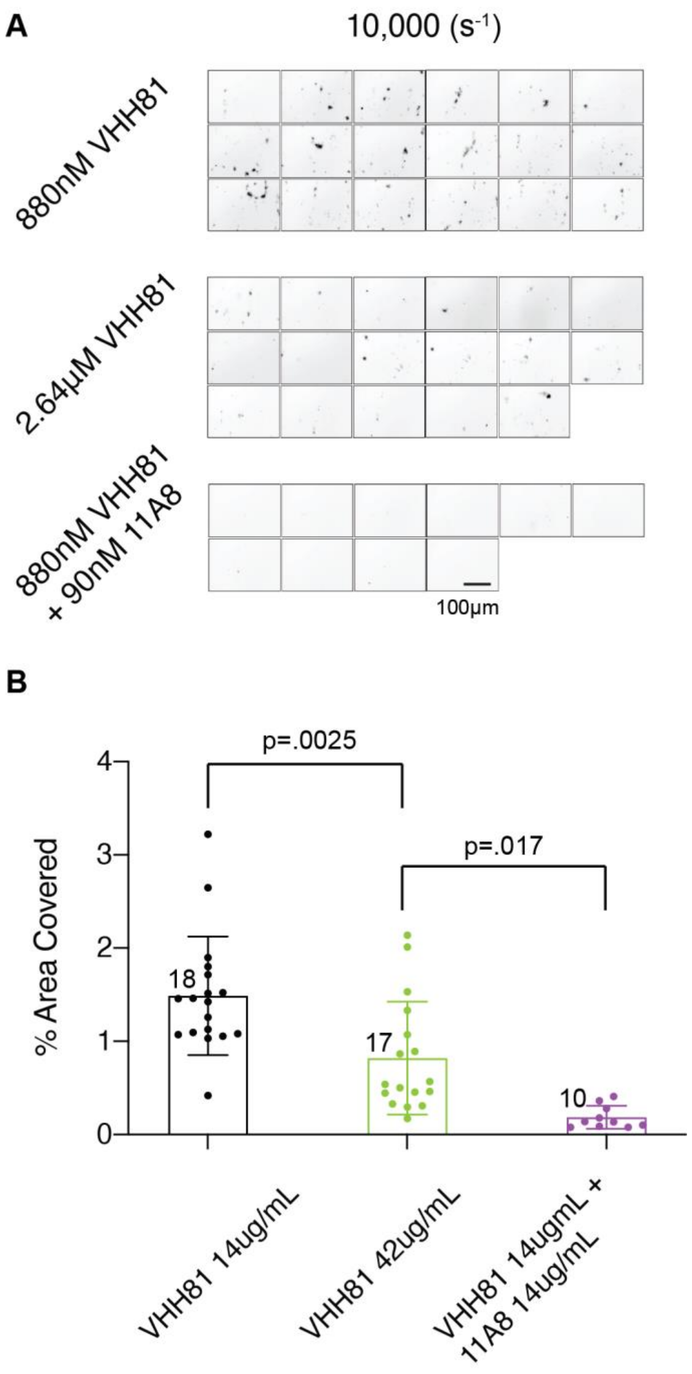

Supplementary Figure 18. Additional parallel-plate flow chamber experiments with VHH81 and the effects of direct inhibition of GPIbα by the anti-GPIbα monoclonal antibody 11A8. (A) Representative flow chamber images of platelet adhesion on collagen coated substrate with additional VHH81 or anti GPIbα LBD antibody 11A8. Scale bar is 100 µm. (B) Data presented in histogram is mean ± standard deviation. Number of images used for analysis is denoted next to the histogram. Comparison between area covered was analyzed with a 1-way ANOVA with Tukey's multiple comparison correction, $F(2, 42) = 18.57$. P-values are denoted above the comparison bars.
