# Supplementary material for: Activation of von Willebrand factor via mechanical unfolding of its discontinuous autoinhibitory module
Source: Nat Commun. 2021 Apr 21;12:2360. doi: 10.1038/s41467-021-22634-x (PMC8060278; doi:10.1038/s41467-021-22634-x)
Supplement: Supplementary file 2 — Description of Additional Supplementary Files [file 41467_2021_22634_MOESM2_ESM.docx]

**Description of Additional Supplementary Files**

File Name: **Supplemental Video 1**

Description: A movie is shown of the final refined electron density maps calculated using phenix with 2mFo-DFc coefficients, where Fo and Fc are the observed and calculated structure factors, respectively. The map was displayed using Pymol (grey) at a contour level of 1.6 r.m.s. and the refined model is shown as sticks. At the top in green is the A1 domain in the region of the Cys1272-Cys1458 disulfide bond and below in cyan is the nanobody.

File Name: **Supplemental Video 2**

Description: An animated cartoon illustrating activation of A1 to bind GPIbα by mechanical disruption of the AIM, as well as the stabilization of the AIM. The animation is based on crystal structures of the AIM-A1/VHH81 complex and the A1/GPIbα-LBD complex.
