# Supplementary material for: Activation of von Willebrand factor via mechanical unfolding of its discontinuous autoinhibitory module
Source: Nat Commun. 2021 Apr 21;12:2360. doi: 10.1038/s41467-021-22634-x (PMC8060278; doi:10.1038/s41467-021-22634-x)
Supplement: Supplementary file 5 — Reporting Summary [file 41467_2021_22634_MOESM5_ESM.pdf]

nature research

Corresponding author(s): Renhao Li, X. Frank Zhang

Last updated by author(s): Mar 5, 2021

Reporting Summary

Nature Research wishes to improve the reproducibility of the work that we publish. This form provides structure for consistency and transparency in reporting. For further information on Nature Research policies, see our Editorial Policies and the Editorial Policy Checklist.

Statistics

For all statistical analyses, confirm that the following items are present in the figure legend, table legend, main text, or Methods section.

n/a	Confirmed	
☐	☒	The exact sample size (n) for each experimental group/condition, given as a discrete number and unit of measurement
☐	☒	A statement on whether measurements were taken from distinct samples or whether the same sample was measured repeatedly
☐	☒	The statistical test(s) used AND whether they are one- or two-sided<br>*Only common tests should be described solely by name; describe more complex techniques in the Methods section.*
☒	☐	A description of all covariates tested
☐	☒	A description of any assumptions or corrections, such as tests of normality and adjustment for multiple comparisons
☐	☒	A full description of the statistical parameters including central tendency (e.g. means) or other basic estimates (e.g. regression coefficient) AND variation (e.g. standard deviation) or associated estimates of uncertainty (e.g. confidence intervals)
☐	☒	For null hypothesis testing, the test statistic (e.g. F, t, r) with confidence intervals, effect sizes, degrees of freedom and P value noted<br>*Give P values as exact values whenever suitable.*
☒	☐	For Bayesian analysis, information on the choice of priors and Markov chain Monte Carlo settings
☒	☐	For hierarchical and complex designs, identification of the appropriate level for tests and full reporting of outcomes
☒	☐	Estimates of effect sizes (e.g. Cohen's d, Pearson's r), indicating how they were calculated

Our web collection on statistics for biologists contains articles on many of the points above.

Software and code

Policy information about availability of computer code

Data collection	Diffraction data was collected on beamline I04 and Diamond Light Source at the Harwell Science and Innovation Campus in Oxfordshire, United Kingdom.
Data analysis	Aggrolink8 (v1.3.98)<br>ForteBio Data Acquisition (v11.1.1.19)<br>ForteBio Data Analysis HT (v11.1.1.39)<br>GraphPad Prism (v8.4)<br>Phaser (v2.8.3)<br>Buccaneer (1.6.5)<br>COOT(0.8.9.2)<br>REFMAC(5.8.0158)<br>CCP4 (v7.0)<br>FIJI (v1.0)<br>Pymol (v2.3.4)

For manuscripts utilizing custom algorithms or software that are central to the research but not yet described in published literature, software must be made available to editors and reviewers. We strongly encourage code deposition in a community repository (e.g. GitHub). See the Nature Research guidelines for submitting code & software for further information.

Data

Policy information about availability of data

All manuscripts must include a data availability statement. This statement should provide the following information, where applicable:

- Accession codes, unique identifiers, or web links for publicly available datasets
- A list of figures that have associated raw data
- A description of any restrictions on data availability

The data that support the findings of this study are available from the corresponding authors upon reasonable request. Protein coordinates and structure factors have been deposited in the RCSB Protein Data Bank under code 7A6O at 10.2210/pdb7A6O/pdb
Structure 1AUQ is available from the Protein Data Bank at 10.2210/pdb1AUQ/pdb
Structure 1SQ0 is available from the Protein Data Bank at 10.2210/pdb1SQ0/pdb

Field-specific reporting

Please select the one below that is the best fit for your research. If you are not sure, read the appropriate sections before making your selection.

- [x] Life sciences
- [ ] Behavioural & social sciences
- [ ] Ecological, evolutionary & environmental sciences

For a reference copy of the document with all sections, see nature.com/documents/nr-reporting-summary-flat.pdf

Life sciences study design

All studies must disclose on these points even when the disclosure is negative.

Sample size	No statistical methods were used to predetermine sample size. For platelet aggregation and ELISA, triplicate measurements were performed as is standard for these measurements. For flow-chamber experiments, at least 12 images were used to determine surface coverage, although up to 25 images were captured to include the entire chamber where platelets could adhere to the coated surface. Single molecule force-spectroscopy measurements were made with hundreds of traces collected per condition or protein tested.
Data exclusions	No data was excluded.
Replication	The purified proteins in this study have been repeatedly produced and purified from stable expressing cell lines or bacterial glycerol stocks. Measurements of platelet aggregation and platelet adhesion were repeated with different blood donors with similar results seen throughout. ELISA assays were performed in at least triplicate and repeated at least twice with similar results. BLI experiments were performed twice with similar results observed for amplitude of sensorgrams and fitting parameters for the dilution series tested. Single molecule force-spectroscopy measurements were made with hundreds of traces collected per condition or protein tested at several loading rates.
Randomization	No randomization was specifically applied for this study. Experiments had to be performed with prior knowledge of the protein being tested or randomization was irrelevant with regards to biochemical assays.
Blinding	The investigators were not blinded during experiments or assessment. As the overwhelming majority of the experiments performed are objective measurements obtained from various instruments. Measurement of platelet adhesion was not blinded, but all data was subject to very similar masking parameters to determine coverage area.

Reporting for specific materials, systems and methods

We require information from authors about some types of materials, experimental systems and methods used in many studies. Here, indicate whether each material, system or method listed is relevant to your study. If you are not sure if a list item applies to your research, read the appropriate section before selecting a response.

Materials & experimental systems

n/a	Involved in the study	
[ ]	[x]	Antibodies
[ ]	[x]	Eukaryotic cell lines
[x]	[ ]	Palaeontology and archaeology
[x]	[ ]	Animals and other organisms
[ ]	[x]	Human research participants
[x]	[ ]	Clinical data
[x]	[ ]	Dual use research of concern

Methods

n/a	Involved in the study	
[x]	[ ]	ChIP-seq
[x]	[ ]	Flow cytometry
[x]	[ ]	MRI-based neuroimaging

Antibodies

Antibodies used	Anti-His tag Antibody 4E3D10H2/E3 (Thermofisher, Cat. MA1-135),<br>11A8 (PMID:28692141, produced in the Renhao Li Lab)<br>6G1 (PMID: 10607699, Berndt Lab, purified in the Renhao Li Lab)<br>CR1 (PMID: 10607699, Berndt Lab, purified in the Renhao Li Lab)<br>HRP-conjugated anti-mouse secondary antibody (Santa Cruz Biotechnology, Cat. sc2005)<br>HRP-conjugated anti-VHH monoclonal antibody 96A3F5 (Genscript Cat. A01860)<br>IRDye 800CW Goat anti-Mouse IgG (Licor Cat. 926-32210)
Validation	4E3D10H2/E3 was validated by western blot, IP, and ELISA by the manufacturer, MA1-135 detects both N-terminal and C-terminal His-tagged proteins.<br>11A8 has been validated as a specific human GPIbα-A1 binding site inhibitor by ELISA and flow cytometry using human platelets and CHO cells expressing human GPIb-IX complex by our lab (PMID: 28692141, 29203584, 31233025, 31030883)<br>6G1 and CR1 were validated via western-blot (PMID: 10607699, and repeated by our lab) and ELISA (this paper) for their specificity- 6G1 reacts with reduced and non-reduced VWF and VWF fragments containing the epitope from 1461-1472 by western blot. CR1 reacts only with non-reduced VWF and VWF fragments containing at least residues 1238-1472, and does not react with reduced VWF or VWF fragment by western blot.<br>sc2005 was validated by western blot for detecting mouse primary antibody 300-3 (Santa Cruz Biotechnology) bound to humanTenascin-C.<br>96A3F5 mAb is specific for Camelid sdAb (Llama and Camel). The monoclonal antibody has no cross-reactivity with mouse, rat, rabbit, chicken, goat or human immunoglobulins by western blot and ELISA.<br>IRDye 800CW Goat anti-Mouse IgG. Based on ELISA and flow cytometry, this antibody reacts with the heavy and light chains of mouse IgG1, IgG2a, IgG2b, and IgG3, and with the light chains of mouse IgM and IgA. This antibody was tested by dot blot and and/or solid-phase adsorbed for minimal cross-reactivity with human, rabbit, goat, rat, and horse serum proteins, but may cross-react with immunoglobulins from other species. The conjugate has been specifically tested and qualified for Western blot applications.

Eukaryotic cell lines

Policy information about cell lines

Cell line source(s)	Expi293F cells were purchased from ThermoFisher, A14527.
Authentication	Expi293F cells were not authenticated. Cells were used to produce recombinant proteins and no cell-based assays were used.
Mycoplasma contamination	Cell lines were not tested for mycoplasma contamination.
Commonly misidentified lines<br>(See ICLAC register)	No commonly misidentified cell lines were used.

Human research participants

Policy information about studies involving human research participants

Population characteristics	Blood samples were collected from healthy donors of both sexes and various ethnicities (Hispanic, White, Asian) and ages (22-50)
Recruitment	Human subjects were recruited from various labs on the Emory campus. As only human platelet activity was measured in response to various agonists/antagonists it is unlikely that any self-selection bias is present, as normal, healthy volunteers with normal, healthy blood would be needed to evaluate platelet activity.
Ethics oversight	The Emory University IRB has reviewed and approved the use of human subjects (IRB00006228).

Note that full information on the approval of the study protocol must also be provided in the manuscript.
